# Persistent paramagnons in high-temperature infinite-layer nickelate superconductors

**DOI:** 10.1038/s41467-026-73083-3

**Published:** 2026-05-20

**Authors:** Yujie Yan, Ying Chan, Xunyang Hong, S. Lin Er Chow, Zhaoyang Luo, Yuehong Li, Tianren Wang, Yuetong Wu, Izabela Biało, Nurul Fitriyah, Saurav Prakash, Xing Gao, King Yau Yip, Qiang Gao, Xiaolin Ren, Jaewon Choi, Ganesha Channagowdra, Jun Okamoto, Xingjiang Zhou, Zhihai Zhu, Liang Si, Mirian Garcia-Fernandez, Ke-Jin Zhou, Hsiao-Yu Huang, Di-Jing Huang, Johan Chang, A. Ariando, Qisi Wang

**Affiliations:** 1 Department of Physics, The Chinese University of Hong Kong, Shatin, Hong Kong, China; 2 State Key Laboratory of Quantum Information Technologies and Materials, The Chinese University of Hong Kong, Shatin, Hong Kong, China; 3 Physik-Institut, Universität Zürich, Zürich, Switzerland; 4 Department of Physics, Faculty of Science, National University of Singapore, Singapore, Singapore; 5 Department of Physics, Faculty of Science and Technology, Universitas Airlangga, Surabaya 60115, East Java, Indonesia; 6 Beijing National Laboratory for Condensed Matter Physics, Institute of Physics, Chinese Academy of Sciences, Beijing, China; 7 Diamond Light Source, Harwell Campus, Oxfordshire, Didcot OX11 0DE, United Kingdom; 8 Department of Physics, Korea Advanced Institute of Science and Technology, Daejeon, Republic of Korea; 9 National Synchrotron Radiation Research Center, Hsinchu, Taiwan; 10 School of Physics, Northwest University, Xi’an, China

**Keywords:** Superconducting properties and materials, Electronic properties and materials

## Abstract

The recent discovery of high-temperature superconductivity in hole-doped SmNiO_2_, exhibiting the record-high transition temperature *T*_*c*_ among infinite-layer (IL) nickelates, has opened a new avenue for exploring design principles of superconductivity. Experimentally determining the electronic structure and magnetic interactions in this new system is crucial to elucidating the mechanism behind the enhanced superconductivity. Here, we report a Ni *L*-edge resonant inelastic x-ray scattering (RIXS) study of superconducting Sm-based IL nickelate thin films Sm_1−*x*−*y*_Eu_*x*_Ca_*y*_NiO_2_ (SECNO). Dispersive paramagnonic excitations are observed in both optimally and overdoped SECNO samples, supporting a spin-fluctuation-mediated pairing scenario. However, despite the two-fold enhancement of *T*_*c*_ in the Sm-based nickelates compared to their Pr-based counterparts, the effective exchange coupling strength is reduced by approximately 20%. This behavior contrasts with hole-doped cuprates, where magnetic interactions correlate positively with *T*_*c*_, highlighting essential differences in their superconducting mechanisms.

## Abstract (web summary)

The authors report a resonant inelastic x-ray scattering study of superconducting Sm-based infinite-layer nickelate thin films. Despite the two-fold enhancement of *T*_*c*_ in the Sm-based nickelates compared to their Pr-based counterparts, they find that the effective in-plane exchange coupling strength is reduced by approximately 20%.

## Introduction

The discovery of superconductivity in nickelate materials—featuring both square-planar and Ruddlesden-Popper (RP) structures—has unveiled a new family of unconventional high-temperature superconductors^1–4^. With a similar crystal structure and nominal *d*^9^ electronic configuration, the infinite-layer (IL) nickelates have been proposed as analogs to the cuprate superconductors^5,6^. In fact, similar antiferromagnetic excitations and a hole-like Fermi pocket with $3{d}_{{x}^{2}-{y}^{2}}$ character are observed in IL-nickelate and cuprate superconductors^7–10^. Strange metal behavior around optimally doping is another commonality^11,12^. However, distinctions in their electronic behaviors have also been revealed. For example, while the parent cuprates are considered as charge-transfer insulators, the IL nickelates are closer to the Mott-Hubbard regime, due to reduced ligand-oxygen hybridization^6,13–15^. In addition to the correlated cuprate-like hole pocket from Ni ${d}_{{x}^{2}-{y}^{2}}$ band, a three-dimensional electron pocket is observed at the Brillouin zone corner^8–10^, though the role of multi-orbital physics remains uncertain^16,17^. Moreover, while hole-doped cuprates exhibit clear signatures of charge inhomogeneity^18,19^, the existence of charge order in IL nickelates is still controversial^20–25^.

Another contrast is the role played by the rare-earth ions in determining the low-energy physical properties. Whereas rare-earth elements do not exhibit a pronounced influence on the electronic structure of most cuprates, significant hybridization between rare-earth 5*d* and Ni 3*d* bands has been observed in IL nickelates^26^. Self-doping from the rare-earth 5*d* states into the NiO_2_ planes has presumably led to a metallic state and the absence of magnetic order in the parent compounds^1,11,27^. Experiments further revealed distinct electronic behaviors among IL nickelates with different rare-earth elements^28–31^. For example, while Sr-doped LaNiO_2_ and PrNiO_2_ exhibit strongly anisotropic upper critical field *H*_*c*2_^31^, an unexpected isotropic *H*_*c*2_ is revealed in (Nd,Sr)NiO_2_^31,32^. However, the origin of these differences, and their implications for superconductivity, remain subjects of intense debate^33–35^.

Recently, superconductivity was realized in high-crystallinity, phase-pure, hole-doped Sm-based IL nickelate thin films^36,37^, which exhibit an optimal superconducting onset temperature *T*_*c*_ approaching 40 K with zero-resistance state at *T*_*c*,0_ ≈ 30 K—the highest reported among all IL nickelates to date. The nearly two-fold increase in *T*_*c*_ compared to hole-doped (Nd/La/Pr)NiO_2_ systems highlights Sm-based compounds as a promising platform for understanding the mechanism of superconductivity in these materials, especially the role of the rare-earth elements. While some studies suggest that reduced interlayer spacing^37,38^ or smaller in-plane lattice constants^39,40^ may enhance *T*_*c*_, the associated changes in electronic structure and magnetic interactions remain unclear—yet are essential for uncovering the microscopic mechanism of superconductivity.

**Fig. 1:**
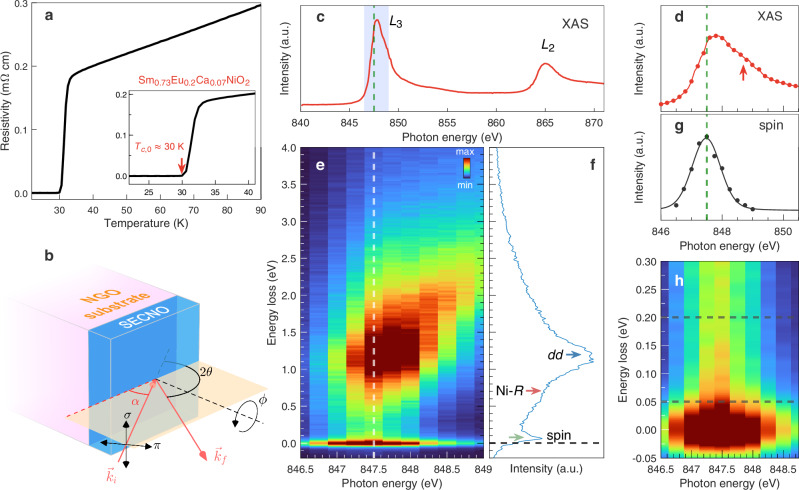
Resistivity measurement, XAS, and RIXS intensity map for optimally doped SECNO. **a** The temperature-dependent resistivity for the OP SECNO film used for RIXS measurements. **b** Scattering geometry of the RIXS experiment. The wave vectors of the incident and scattered photons (${\vec{k}}_{i}$ and ${\vec{k}}_{f}$) define the scattering angle 2*θ*. **c** XAS spectrum across the Ni *L*_3_ (~847.5 eV) and *L*_2_ (~865 eV) resonances measured at 30 K using total electron yield. **d** Normal-incidence XAS across the Ni *L*_3_ edge. An additional shoulder (red arrow) is observed at ~1 eV above the main peak. **e** RIXS intensity as a function of incident photon energy and energy loss, measured at 30 K with an incident angle *α* = 118. 3^∘^ and azimuthal angle *ϕ* = 0^∘^, corresponding to **Q** = (0.35, 0, 0.31). The range of incident energy is indicated by the blue-shaded area in (**c**). **f** RIXS spectrum taken at the resonance energy (green dashed lines in (**c**, **d**, **g**); white dashed line in (**e**)). Elastic scattering has been subtracted to enhance the visibility of low-energy features. Blue, red and green arrows indicate excitations around 1.2 eV, 0.7 eV and 0.1 eV, respectively. **g** Resonant profile of the ~0.1 eV excitation (intensity integrated between 0.05 and 0.2 eV). **h** Enlarged view of the low-energy region in (**e**). Gray dashed lines mark the intensity integration range for (**g**). a.u., arbitrary units.

In this study, we employ resonant inelastic x-ray scattering (RIXS) at Ni *L*-edge to investigate the electronic and magnetic excitations in superconducting Sm_1−*x*−*y*_Eu_*x*_Ca_*y*_NiO_2_ (SECNO) thin films. An optimally doped (OP) sample Sm_0.73_Eu_0.2_Ca_0.07_NiO_2_ (nominal doping *p* = 0.19) with a superconducting onset temperature *T*_*c*,onset_ ≈ 35 K (Fig. 1a), and a non-superconducting overdoped (OD) sample Sm_0.53_Eu_0.4_Ca_0.07_NiO_2_ (nominal doping *p* = 0.31) are studied (Supplementary Note 1 and ref. ^36^). To distill the key ingredients for superconductivity in IL nickelates, comparative measurements are performed on an optimally doped Pr_0.8_Sr_0.2_NiO_2_ (PSNO) thin film with *T*_*c*,onset_ ≈ 9 K ^39^. We observe dispersive paramagnonic excitations in SECNO, with a bandwidth of approximately 100 meV, reduced by  ~20% compared to PSNO. The persistence of robust magnetic correlations with large exchange couplings across these IL nickelate systems supports the scenario in which superconducting pairing is primarily mediated by spin fluctuations^41–46^. Nevertheless, the significantly enhanced *T*_*c*_ in Sm-based nickelates deviates from the cuprate-like trend, where larger exchange couplings correlate positively with higher *T*_*c*_^47,48^. Our findings thus suggest that additional factors, such as the multi-band electronic structure, enhanced three-dimensionality, and Kondo-like hybridization involving rare-earth orbitals, need to be considered to fully capture the microscopic mechanism underlying superconductivity in IL nickelates.

## Results

Due to the difficulties in achieving phase-pure undoped SmNiO_2_ thin film^49^, we studied optimally and overdoped SECNO samples free from Ruddlesden-Popper-type extended defects^36^. X-ray absorption spectrum (XAS) of the optimally doped SECNO thin film across the Ni *L*_3_ and *L*_2_ edges is shown in Fig. 1c. A shoulder—due to doped holes in the Ni ${d}_{{x}^{2}-{y}^{2}}$ orbital^50^—appears about 1 eV above the Ni *L*_3_ peak (Fig. 1d). To probe the electronic excitations, we performed RIXS measurements with the incident photon energy varied across the Ni *L*_3_ edge—indicated by the blue-shaded area in Fig. 1c. Fluorescence dominated RIXS intensities are observed at energies of 2.5 eV and above (see Fig. 1e). The *dd* excitations centered around 1.2 eV resemble those observed in hole-doped Nd_1−*x*_Sr_*x*_NiO_2_^50^. The excitation at ~0.7 eV (Fig. 1f) is attributed to hybridization between Ni 3*d* and rare-earth 5*d* orbitals^26^, evidencing occupied rare-earth 5*d* states below the Fermi level in the superconducting regime. Overall, the key features of the *dd* excitations are found similar to other IL nickelates^33^. A low-energy excitation is identified around 0.1 eV, which is most prominent at an incident energy of 847.5 eV (Fig. 1h). The resonant behavior demonstrates its electronic origin (Fig. 1g, h), arising from valence-state transitions of Ni 3*d* orbitals.

**Fig. 2:**
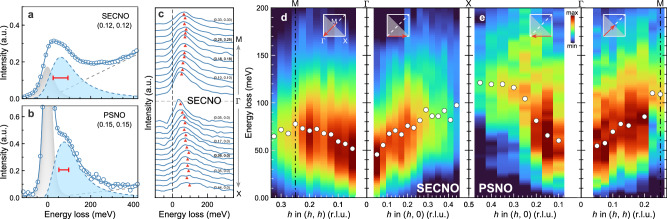
Paramagnonic excitations in optimally doped SECNO and PSNO. Representative raw RIXS spectra of OP **a** SECNO and **b** PSNO samples. The blue solid line denotes the fitting result with components indicated by shaded areas and dashed lines: elastic scattering (gray), paramagnon excitation (light blue), and high-energy background (gray dashed line). The red horizontal bars denote the instrumental energy resolution. To reduce elastic scattering, a scattering angle 2*θ* = 90^∘^ is used in (**a**). **c** Momentum-dependent RIXS spectra of OP SECNO. Elastic and background scattering has been subtracted. Red arrows indicate the position of the maximum intensity of the fitted paramagnonic excitations. The gray vertical arrows indicate the scan directions of the in-plane momentum transfer, and a complete list of wave vector for each spectrum is provided in Supplementary Table 2. RIXS intensity map measured along momentum directions (*h*, 0) and (*h*, *h*) in OP **d** SECNO and **e** PSNO. White dots indicate the peak energy (${\omega }_{\max }$) of the magnetic excitation mode extracted from fits (see main text). Black dash-dot lines mark the antiferromagnetic zone boundary. The scan directions of the in-plane momentum are illustrated in the insets. a.u., arbitrary units.

To establish the nature of this low-energy excitation, we performed RIXS measurements (Fig. 1b) along the high-symmetry (*h*, 0) and (*h*, *h*) momentum directions at the Ni *L*_3_ edge (847.5 eV). Each spectrum is fitted with a multi-component model—see Fig. 2a, b. The quasi-elastic scattering is described by a Voigt profile (shown in gray); the low-energy excitation mode topping around 100 meV is modeled as a damped harmonic oscillator (DHO) convoluted with the instrumental resolution (blue shaded area); the background, primarily arising from the particle-hole continuum, is approximated by a quadratic function (gray dashed line). The influence of potential contributions from phonon modes is discussed in Supplementary Note 2B. In this fashion, we extract the momentum dependence of the low-energy mode, as summarized in Fig. 2c, d.

With increasing momentum—along (*h*, 0) and (*h*, *h*) directions—the excitation mode shifts to higher energy (Fig. 2c, d), in agreement with the characteristic dispersion of a square-lattice antiferromagnetic paramagnon. The broad energy width (~170 meV)—significantly exceeding the resolution—further supports our assignment of this excitation as a paramagnon. As the in-plane momentum transfer increases, the spectral weight is suppressed, and the energy linewidth broadens (see Supplementary Fig. 6). The overall dispersive behavior, the momentum dependence of the spectral weight, and the energy linewidth of the magnetic excitations in OP SECNO are consistent with previous observations of spin excitations in other IL nickelates.^7,33,51^.

**Fig. 3:**
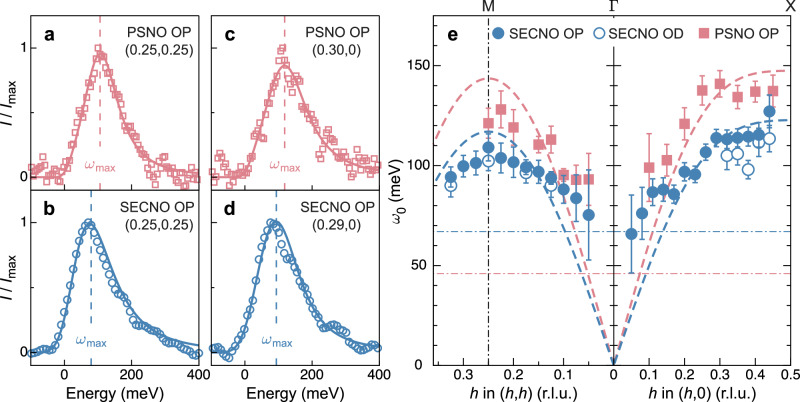
Dispersions of paramagnon in SECNO and PSNO. Paramagnon spectral components for OP PSNO (red) and SECNO (blue) near the antiferromagnetic zone boundaries in the (*h*, *h*) (**a**, **b**) and (*h*, 0) (**c**, **d**) directions. Dashed lines indicate the energy positions of the peak maxima (${\omega }_{\max }$). The intensity *I* is normalized to its peak value ${I}_{\max }$. **e** Paramagnon pole energies (*ω*_0_) extracted from fits to the RIXS spectra for SECNO (blue) and PSNO (red). Error bars represent the fitting uncertainties. Larger error bars near (0, 0) stem from increasing elastic scattering around the specular geometry (See Supplementary Fig. 9). Dashed lines denote fits to an effective Heisenberg model (see main text) for OP SECNO (blue) and PSNO (red). Blue (red) horizontal dashed line indicates the energy resolution (FWHM) for measurements on SECNO (PSNO).

The paramagnon persists in the overdoped SECNO sample. Compared with the optimally doped sample, the overdoped sample shows a likely reduction in both the pole energy *ω*_0_ (undamped excitation energy, see Methods and refs. ^52,53^) and the energy positions of the peak maxima (${\omega }_{\max }$) (see Fig. 3e and Supplementary Fig. 1). While our measurements are limited to these two dopings, this observation is consistent with the doping evolution reported in other nickelate systems^7,54^. Such behavior indicates a gradual softening of spin correlations as additional carriers are introduced and effectively dilutes the spin sites, resembling the observations reported in certain hole-doped cuprates^55–57^. As such, we expect a higher magnon energy in undoped SmNiO_2_.

## Discussion

To gain insight into the influence of rare-earth element on the magnetism, we compare the paramagnon in OP SECNO and PSNO. As shown in Fig. 2d, e, the paramagnon in SECNO displays a comparable but slightly smaller bandwidth. The effective exchange interactions are derived from the paramagnon pole (*ω*_0_) using a linear spin-wave model (see Supplementary Information for details). The reduced zone-boundary energy at (0.25, 0.25) compared to (0.5, 0) reveals the existence of higher-order exchange interactions^58,59^. Given this weak but discernible zone-boundary dispersion, we included both nearest-neighbor (*J*_1_) and next-nearest-neighbor (*J*_2_) exchange couplings:

1 $$H={J}_{1}\mathop{\sum }\limits_{\langle i,j\rangle }{{\bf{S}}}_{i}\cdot {{\bf{S}}}_{j}+{J}_{2}\mathop{\sum }\limits_{\langle i,{i}^{{\prime} }\rangle }{{\bf{S}}}_{i}\cdot {{\bf{S}}}_{{i}^{{\prime} }}$$

where 〈*i*, *j*〉 and $\langle i,{i}^{{\prime} }\rangle$ denote pairs of nearest and next-nearest neighbor spin sites, respectively. The best fit yields *J*_1_ = 47.1 ± 4.7 meV and *J*_2_ = − 2.4 ± 3.6 meV for OP SECNO, and *J*_1_ = 59.2 ± 7.6 meV and *J*_2_ = − 1.6 ± 5.6 meV for OP PSNO, assuming spin *S* = 1/2 and quantum renormalization factor *Z*_*c*_ = 1.18 ^7,58^. Applying the same analysis to the paramagnon dispersion for Nd_0.775_Sr_0.225_NiO_2_^7^, we find a consistently larger magnon bandwidth and stronger exchange coupling compared to OP SECNO (see Supplementary Fig. 7 and Supplementary Table 1).

A ~20% smaller in-plane exchange *J*_∥_ in SmNiO_2_ compared to (Nd/La/Pr)NiO_2_ has been theoretically predicted by first-principles calculations^35^. It is suggested that smaller rare-earth ionic radii enhance the three-dimensional character of the electronic structure. While the exchange interactions remain predominately mediated by the Ni ${d}_{{x}^{2}-{y}^{2}}$ orbitals, the increased three-dimensionality, accompanied by subtle structural modifications^35,60^, may introduce a finite out-of-plane exchange coupling *J*_⊥_. In fact, recent ARPES experiments have observed additional electron Fermi pockets in hole-doped LaNiO_2_^8,9^ and NdNiO_2_^10^. Moreover, the existence of significantly stronger *J*_⊥_ in infinite-layer nickelates compared to that of cuprates has been suggested by a recent RIXS study^61^ (see Supplementary Note 3 for more discussions). Future experiments will be essential to clarify how the electronic and magnetic structures evolve across different rare-earth IL nickelates.

The observation of large exchange couplings across all IL nickelate systems is consistent with theoretical proposals that spin fluctuations play a dominant role in the superconducting pairing^41–46^. Yet, the reduced in-plane exchange coupling *J*_∥_ in SECNO despite its enhanced *T*_*c*_ is markedly different from cuprates, where a positive correlation has been established between *T*_*c*_ and the paramagnon energy scale^47,48^. An absence of proportionality between *T*_*c*_ and in-plane exchange coupling has also been recently observed in PrNiO_2_ grown on different substrates^51^. This difference could possibly originate from the multi-band nature of nickelates. Compared to cuprates, the hybridization between the rare-earth 5*d* and Ni 3*d* states in the IL nickelates renders them relevant to the low-energy physics. The enhanced three-dimensionality may also strengthen Kondo-like coupling effects^17,62,63^, and thereby provide additional pairing strength. In such a Mott-Kondo scenario, the pairing symmetry is predicted to undergo a transition from *d* to *d* + *is* as the Kondo coupling increases^62,63^, which can be examined experimentally in the future. Conversely, a recent polarization-dependent ARPES study suggests that the additional electron Fermi pocket originates from the interstitial *s* states rather than rare-earth 5*d* orbitals^10^. The rare-earth element modifies the electronic and magnetic structures through a chemical pressure effect.

Although the precise role of rare-earth ions in the electronic structure and superconductivity of IL nickelates remains under investigation, our findings indicate that the reduced size of rare-earth ions effectively tunes the magnetic interactions towards a more three-dimensional structure, which may be the key for the enhanced *T*_*c*_. The potentially enhanced contribution of rare-earth electronic states at low energy and the stronger interlayer coupling in IL nickelates, compared to cuprates, could be fundamental for understanding their superconducting mechanisms. Furthermore, the nontrivial relationship between in-plane exchange coupling and *T*_*c*_ revealed by our study points toward alternative design principles for achieving higher *T*_*c*_ in nickelate superconductors, in particular by strengthening the interlayer coupling—for example through epitaxial strain and/or hydrostatic pressure, or via heterostructure engineering.

## Methods

### Film growth

The precursor thin films of Sm_1−*x*−*y*−*z*_Eu_*x*_Ca_*y*_NiO_2_ (SECNO, 6 nm-thick) and Pr_0.8_Sr_0.2_NiO_2_ (PSNO, 8 nm-thick) were grown on NdGaO_3_(110) and SrTiO_3_(001) substrates, respectively, using pulsed laser deposition (PLD), followed by a topotactic reduction process described in refs. ^36,39^. The SECNO and PSNO films were capped with SrTiO_3_ layers of 1 nm and 14 nm thickness, respectively. The optimal *T*_*c*,0_ ≈ 30 K for SECNO is achieved at a hole doping level of *p* ≈ 0.17 − 0.19 ^36^.

### RIXS experiments

Ni *L*-edge RIXS experiments on SECNO and PSNO were carried out at the 41A1 beamline^64^ at the Taiwan Photon Source and I21 beamline^65^ at the Diamond Light Source, respectively. The wave vector **Q** was defined as (*h*, *k*, *l*) = (*ha*/2*π*, *kb*/2*π*, *lc*/2*π*) in reciprocal lattice units (r.l.u.), with *a* = *b* = 3.86 Å and *c* = 3.27 Å for SECNO^36^, and *a* = *b* = 3.905 Å and *c* = 3.37 Å for PSNO ^66^. To maximize the accessible range of in-plane momentum transfer, the 41A1 (I21) spectrometer was positioned at the largest scattering angle of 2*θ* = 150^∘^(154^∘^) unless otherwise indicated, and the sample temperature was set to 30(16) K. The momentum transfer is determined by the scattering angle 2*θ*, light incident angle *α*, and sample azimuthal angle *ϕ* (see Fig. 1b). The values of (*h*, *k*, *l*) and corresponding rotation angles for spectra measured are listed in Supplementary Table 2. The combined energy resolution *δ*, characterized by the full-width-at-half-maximum (FWHM) of the elastic scattering profile of amorphous carbon, was *δ* = 67 meV (46 meV) for measurements at 41A1 (I21). The energy resolutions are smaller than the energy width (2*γ*) and bandwidth (*ω*_0_ at zone boundary) of the paramagnons (see Figs. 2 and 3). The momentum resolution is approximately  ± 0.01 Å^−1^, which corresponds to  ± 0.006 r.l.u. based on the in-plane lattice parameters. The incident x-rays are *π*-polarized to enhance the single spin-flip cross section. The zero energy loss is calibrated using the position of the elastic peak, ensuring that it remains unaffected by minor systematic variations in the absolute photon energy calibration across different beamlines. RIXS intensities were normalized to the weight of the *dd* excitations between 0.5 and 4 eV, as in refs. ^67–70^.

### Fitting of RIXS spectra

Elastic scattering and paramagnon components are fitted using a Voigt and a damped harmonic oscillator function^52,53^, respectively. The width of the elastic profile is set by the instrumental energy resolution *δ* and the damped harmonic oscillator

2 $${\chi }^{{\prime\prime} }({\bf{Q}},\omega )=\frac{\chi^{\prime} ({\bf{Q}})\,\gamma ({\bf{Q}})\,\omega }{{\left({\omega }^{2}-{\omega }_{0}{({\bf{Q}})}^{2}\right)}^{2}+4{\omega }^{2}\gamma {({\bf{Q}})}^{2}}$$

is Gaussian-convoluted with the resolution *δ*, and multiplied by the thermal factor (see Supplementary Information for details). Here, $\chi^{\prime}$ is the real part of the zero frequency susceptibility. *γ* and *ω*_0_ represent the damping factor and the paramagnon pole (undamped excitation energy), respectively. When damping is significant (*γ* → *ω*_0_), the energy position of the peak maximum (${\omega }_{\max }$) is often analysed as well ^47,52,53^.

## Supplementary information

Supplementary Information

## Source data

Transparent Peer Review file

Source Data

## Data Availability

Data supporting the findings of this study are available from the corresponding authors upon request. Source data are provided with this paper.
