# Supplementary material for: Persistent paramagnons in high-temperature infinite-layer nickelate superconductors
Source: Nat Commun. 2026 May 20;17:6645. doi: 10.1038/s41467-026-73083-3 (PMC13381760; doi:10.1038/s41467-026-73083-3)
Supplement: Supplementary file 1 — Supplementary Information [file 41467_2026_73083_MOESM1_ESM.pdf]

Supplementary Information for

Persistent paramagnons in high-temperature infinite-layer nickelate superconductors

Y. Yan *et al.*

Supplementary Note 1. Doping dependence of XAS and RIXS of SECNO

Supplementary Fig. 1a,b displays the Ni L_3-edge XAS spectra of optimally doped $Sm_{0.73}Eu_{0.2}Ca_{0.07}NiO_2$ (SECNO OP) and overdoped $Sm_{0.53}Eu_{0.4}Ca_{0.07}NiO_2$ (SECNO OD) films measured at 30 K. Although direct comparison with the undoped parent compound is not available, the shoulder on the high-energy side of the main peak (indicated by arrows)—previously attributed to doped holes into the Ni $d_{x^2-y^2}$ orbital [1–3]—becomes more pronounced in the overdoped sample. Increased doping leads to further softening of paramagnon energy in the overdoped sample (Supplementary Fig. 1c-fs).

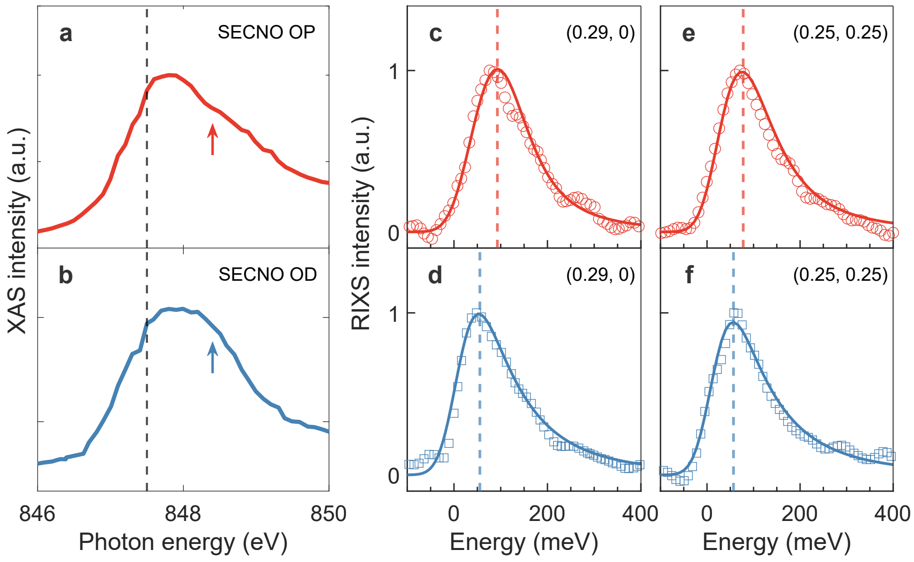

Supplementary Fig. 1. (a,b) Ni L_3-edge XAS measured for (a) optimally doped and (b) overdoped SECNO films at 30 K in normal-incidence geometry using total electron yield. The black dashed line indicates the resonance energy at 847.5 eV. The arrows highlight the shoulder on the high-energy side of the main peak, which becomes more pronounced as doping increases. (c-f) Paramagnon spectral components for OP SECNO (red) and OD SECNO (blue) near the antiferromagnetic zone boundaries along the $(h, 0)$ (c,d) and (h, h) (e,f) directions. Dashed lines indicate the energy positions of the peak maxima ($\omega_{\max}$).

Supplementary Note 2. Fitting and analysis of RIXS spectra

A. Modelling of paramagnonic RIXS cross section

To analyze the spin excitations in doped samples, we adopt a fitting framework based on the damped harmonic oscillator (DHO) model, to describe the imaginary part of the spin susceptibility:

$$\chi''(\mathbf{Q},\omega) = \frac{\chi'(\mathbf{Q})\,\gamma(\mathbf{Q})\,\omega}{\left(\omega^2 - \omega_0(\mathbf{Q})^2\right)^2 + \omega^2\gamma(\mathbf{Q})^2} \tag{1}$$

where $\omega_0(\mathbf{Q})$ denotes the pole energy, $\gamma(\mathbf{Q})$ is the damping factor, and $\chi'(\mathbf{Q})$ represents the real part of the static susceptibility. This formulation captures both the peak position and the linewidth, where the peak energy ω_{max} shifts below the pole energy ω_0 as the damping γ increases [4, 5]. The corresponding RIXS intensity from magnetic scattering is given by:

$$S(\mathbf{Q},\omega) = \frac{\chi''(\mathbf{Q},\omega)}{1 - e^{-\hbar\omega/k_{\mathrm{B}}T}} \tag{2}$$

where $\hbar$ is the reduced Planck constant and k_{B} is the Boltzmann constant.

B. Influence of possible phonon contributions

Previous RIXS studies of IL nickelates have observed phonon excitations around 70 meV with little doping independence [2, 6–8]. Upon hole doping, the energy of magnetic excitations is progressively reduced and eventually overlaps with the phonon energy scale, making it difficult to disentangle phonon and magnetic contributions in raw RIXS spectra. This difficulty is further enhanced for SECNO, where magnetic excitations are located at lower energies. In Ref. 2, phonon components in doped samples are assumed identical to that in the undoped sample. Since undoped $SmNiO_2$ is currently not achievable, to assess the possible influence of phonon contributions, we follow the approach in Ref. 2 and introduce a phonon component at 70 meV in the fitting model for SECNO. The relative phonon intensity is estimated based on previous RIXS studies (e.g., Ref. 2), and allowed to vary by ±30%—similar to the treatment in Ref. 2 for doped samples. In this fashion, we systematically evaluate the uncertainty associated with the phonon contribution and quantify its potential impact on the extracted magnetic energy scale, linewidth, and spectral weight.

As shown in Supplementary Figs. 2 and 3, the inclusion of a phonon component does not alter the extracted key characteristics of magnetic excitations in SECNO. Both the pole (ω_0) and the peak (ω_{max}) energies of the paramagnon remain essentially unchanged within the fitting uncertainties, when including the phonon component. Supplementary Fig. 2 further demonstrates that the linewidth of the magnetic peak ($2\gamma \sim 170$ meV) remains nearly identical over the entire momentum range. From the Brillouin-zone center toward the zone boundary, the magnetic spectral weight decreases gradually, and this trend also remains unchanged.

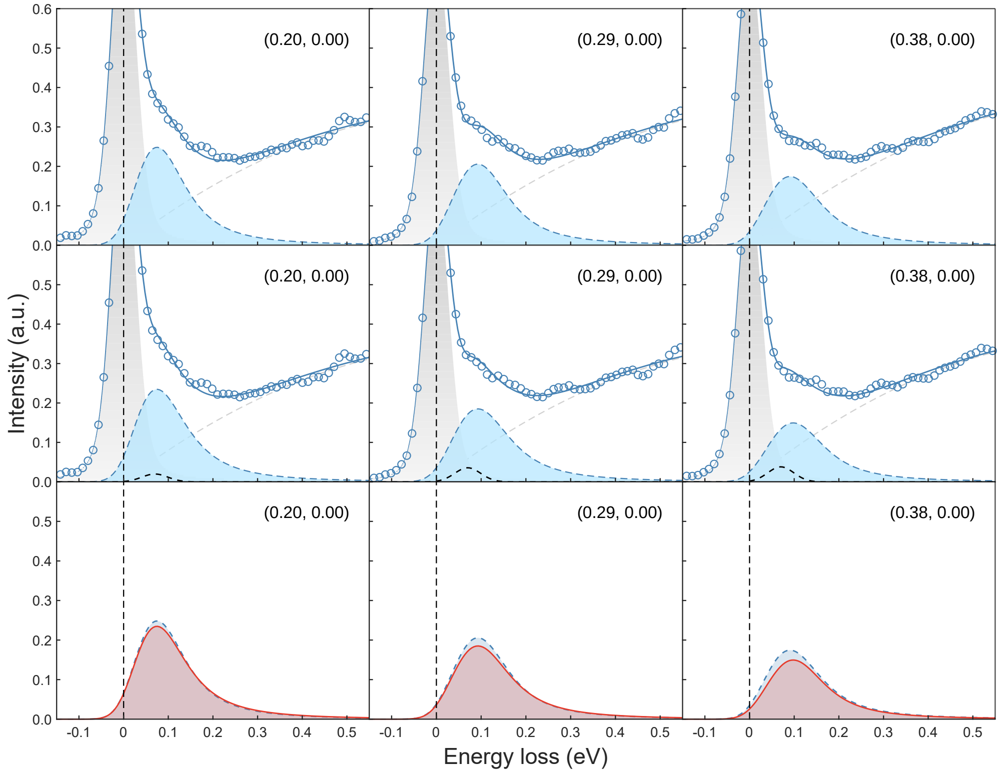

Supplementary Fig. 2. Comparison of analysis with and without phonon contributions for OP SECNO. (Top row) Fittings of spectra using a model without and (middle row) with a phonon component at ∼70 meV, indicated by black dashed curves. (Bottom row) magnetic spectral components extracted from the two fitting models. Red (blue) areas correspond to the model with (without) a phonon component. The dashed vertical lines indicate zero energy loss.

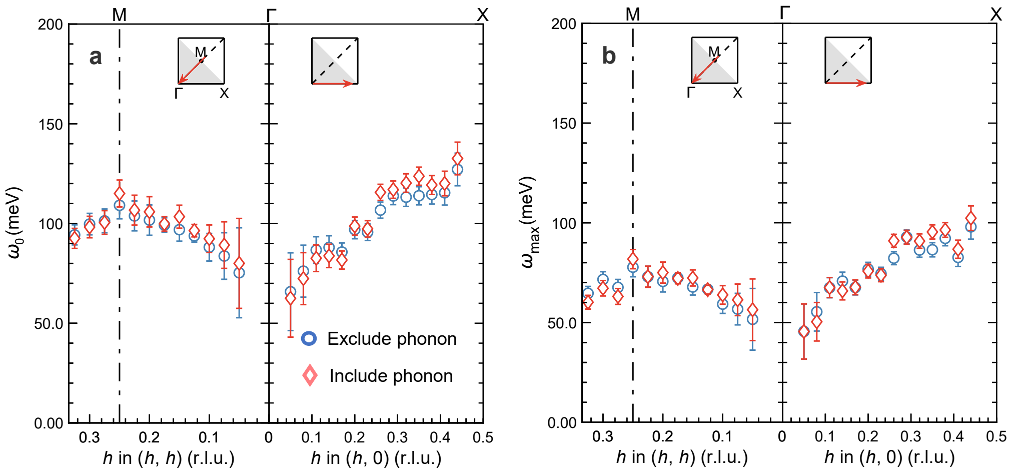

Supplementary Fig. 3. Robustness of paramagnon dispersions against phonon contributions for OP SECNO. Dispersion of (a) the pole energy (ω_0) and (b) the peak maximum ($\omega_{\rm max}$). Results obtained using a fitting model without (blue circles) and with (red diamonds) a phonon component are compared.

For PSNO, the energy of magnetic excitations is overall higher, such that the phonon and magnetic components can be separated in the raw spectra near the zone boundary (Supplementary Fig. 4). Therefore, the phonon component can be directly included in the fitting. Despite this difference, both the magnetic linewidth and spectral weight extracted for SECNO and PSNO are fully comparable (Supplementary Fig. 6a and b). This fact demonstrates that our analysis reliably extracts the key characteristics of the magnetic excitations in both systems.

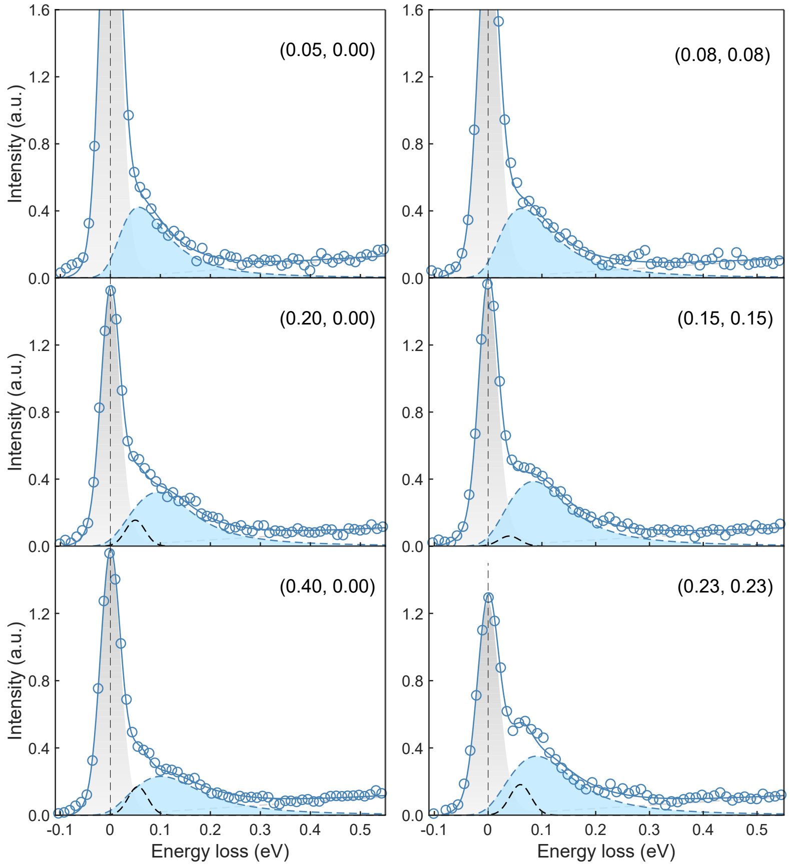

Supplementary Fig. 4. Representative raw RIXS spectra of optimally doped PSNO. The black dashed curves indicate possible phonon components. Shaded areas represent fitting components as described in the manuscript.

C. RIXS measurements at different scattering angles

To maximize the accessible range of in-plane momentum transfer and thereby map out the paramagnon dispersions, most of the RIXS spectra are measured at the largest scattering angle (2θ) of each spectrometer (150° at 41A and 154° at I21). For a consistency check, we conducted additional measurements at $2\theta = 90°$ on SECNO at selective in-plane wave vectors. Such a scattering geometry—with incident light polarized in the scattering plane (π-polarization) and $2\theta = 90°$—is known to minimize the elastic scattering [9]. As demonstrated in Supplementary Fig. 5, as elastic scattering is significantly suppressed, the low-energy part becomes dominated by the magnetic mode. Meanwhile, the extracted paramagnon components remain consistent across two scattering angles, further justifying the reliability of

our analysis. Note that within the accessible range of 2θ, the out-of-plane momentum (l) is restricted to a limited range. For example, at $(0.12, 0.12, l)$, l varies from 0.29 to 0.41 r.l.u. for 2θ between 90° and 150°. In contrast, the examination of any out-of-plane modulation would require a significantly broader coverage of l.

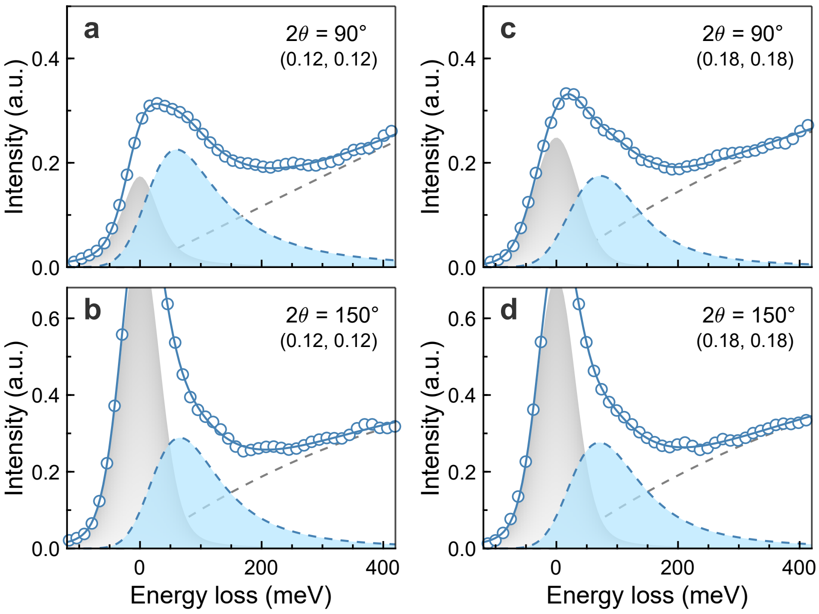

Supplementary Fig. 5. Comparison of raw RIXS spectra measured at different scattering angles for OP SECNO. (a,b) RIXS spectra at $(0.12, 0.12, l)$ measured at (a) $2\theta = 90°$ ($l = 0.28$) and (b) $2\theta = 150°$ ($l = 0.41$). (c,d) RIXS spectra at $(0.18, 0.18, l)$ measured at (c) $2\theta = 90°$ ($l = 0.24$) and (d) $2\theta = 150°$ ($l = 0.38$). Shaded areas and dashed lines denote fitting components as described in the manuscript.

D. Spectral moment analysis

RIXS probes the imaginary part of the dynamical spin susceptibility, $\chi''(\mathbf{q}, \omega)$, with the spectral function directly reflects the overall energy distribution, total spectral weight, and energy broadening of the magnetic excitations. Based on this property, a direct comparison between the results obtained from damped harmonic oscillator (DHO) fitting and a model-independent spectral moment analysis provides an independent validation of the physical reliability of the DHO description, allowing us to assess whether the model successfully captures the essential characteristics of the magnetic excitation spectrum. The spectral moments are defined as $A_n(\mathbf{q}) = \int \omega^n A(\mathbf{q}, \omega) d\omega$ [10], where $A(\mathbf{q}, \omega)$ denotes the magnetic excitation spectrum. For a bosonic response function, the zeroth moment $A_0(\mathbf{q})$ corresponds to the total spectral weight, while the first moment $A_1(\mathbf{q}) = \int \omega A(\mathbf{q}, \omega) d\omega \,/A_0(\mathbf{q})$ defines the average energy. To quantify the spread of the spectrum around average energy, the second spectral moment is defined as $A_2(\mathbf{q}) = \int (\omega - A_1(\mathbf{q}))^2 A(\mathbf{q}, \omega) d\omega / A_0(\mathbf{q})$. The quantity $\sqrt{A_2(\mathbf{q})}$ therefore provides a measure of the spectral broadening around the average energy and can be directly associated with the damping parameter γ of the magnetic excitations.

Supplementary Fig. 6 shows the first three spectral moments and the damping parameter γ extracted for SECNO and PSNO using the DHO model. In both systems, the spectral moment analysis is fully consistent with the DHO

fitting results, with the first and second moment satisfying $A_1(q) \approx \omega_0(q)$ and $\sqrt{A_2(q)} \approx \gamma_q$, respectively.

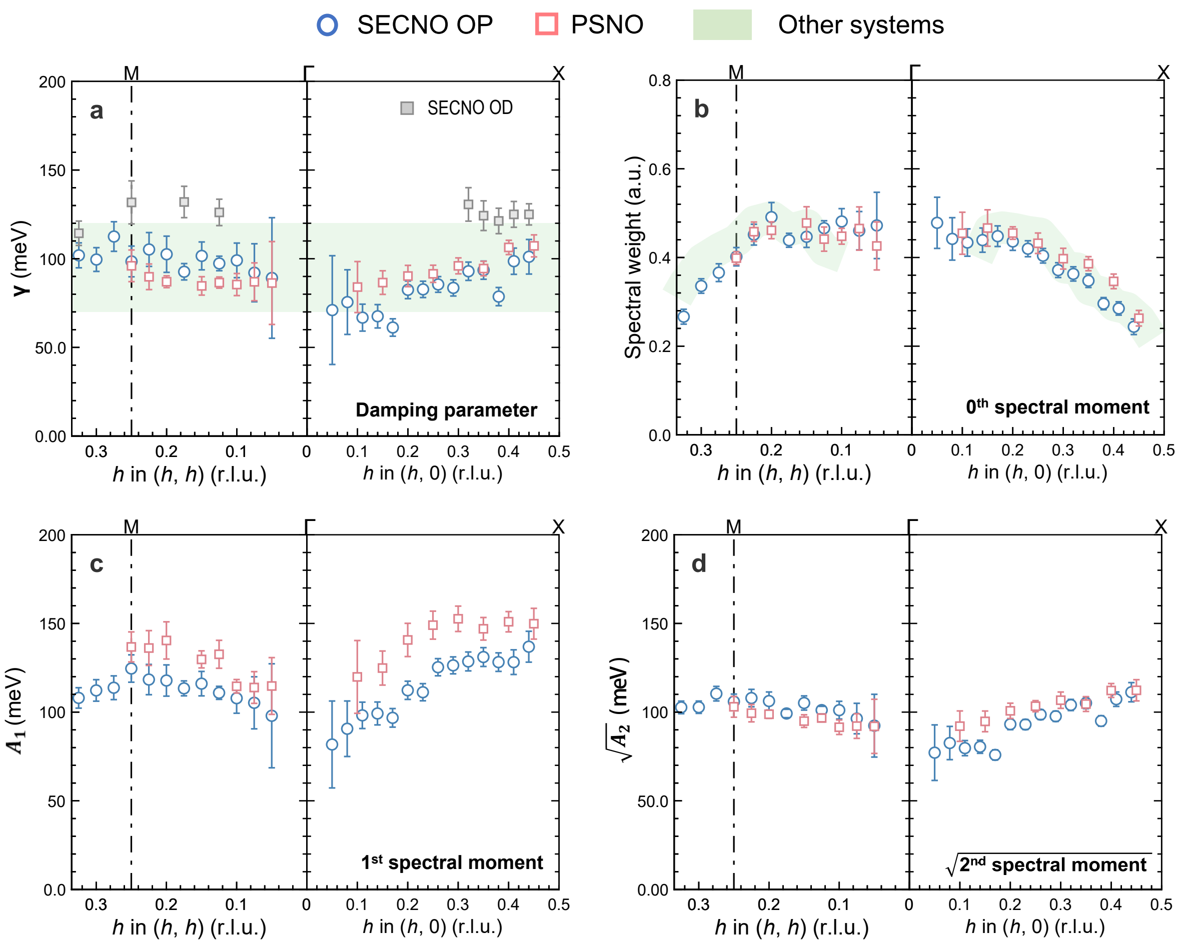

Supplementary Fig. 6. Spectral moment analysis for optimally doped SECNO (blue) and PSNO (red). (a) Damping parameter γ_q extracted from the DHO fitting for optimally doped SECNO and PSNO, the light green shaded region indicates the range of γ values reported for other infinite-layer nickelate systems around optimal doping [2, 6, 8]. Gray squares denote results for overdoped SECNO, which show an overall larger damping parameter γ than the optimally doped sample. (b–d) $A_0(q)$, $A_1(q)$ and $\sqrt{A_2(q)}$, respectively. The zeroth moment $A_0(q)$ in (b) is normalized to the intensity of the *dd* excitations. The light green shaded line (b) outlines the trend of spectral weight in optimally doped NSNO [2].

Supplementary Note 3. Paramagnon dispersions in hole-doped (Sm/Pr/Nd)NiO$_2$

To evaluate the magnetic interaction strengths, we use an effective Heisenberg Hamiltonian to model the paramagnon dispersions

$$H = J_1^{\text{eff}} \sum_{\langle i,j \rangle} \mathbf{S}_i \cdot \mathbf{S}_j + J_2^{\text{eff}} \sum_{\langle i,i' \rangle} \mathbf{S}_i \cdot \mathbf{S}_{i'} \tag{3}$$

where $\langle i, j\rangle$ and $\langle i, i'\rangle$ denote pairs of nearest- and next-nearest-neighbor spin sites, and $\mathbf{S}_i$ denotes the spin-1/2 operator on lattice site i. The paramagnon dispersion is given by the linear spin-wave theory

$$\hbar\omega_q = 2Z_c\sqrt{A_q^2 - B_q^2}, \tag{4}$$

where

$$A_q = J_1^{\text{eff}} - J_2^{\text{eff}}\left[1 - \cos(2\pi h)\cos(2\pi k)\right], \qquad B_q = \frac{1}{2}J_1^{\text{eff}}\left[\cos(2\pi h) + \cos(2\pi k)\right], \tag{5}$$

and the renormalization factor $Z_c = 1.18$ is used for spin-1/2 systems [11, 12]. Due to their damped nature, the energy positions of the paramagnon intensity maxima (ω_{max}) are lower than the pole energies (ω_0) [4, 5]. As illustrated in Supplementary Fig. 7, both characteristic energies are reduced in OP SECNO compared to PSNO. The linear spin-wave model fitting parameters of both ω_0 and ω_{max} are summarized in Supplementary Table 1. A consistent ∼20% reduction in the in-plane effective nearest-neighbor exchange coupling (J_1^{eff}) is inferred for OP SECNO compared to PSNO. Applying the same analysis to the paramagnon dispersion for $Nd_{0.775}Sr_{0.225}NiO_2$ [2], we find a consistently larger magnon bandwidth and stronger exchange coupling compared to OP SECNO (see Supplementary Fig. 7 and Supplementary Table 1).

A recent RIXS study suggests that the out-of-plane exchange coupling $J_\perp$ in IL nickelates may be significantly stronger than that in cuprates [7]. To assess the degree of three-dimensionality in magnetic interactions, we therefore employ a Heisenberg Hamiltonian that explicitly includes $J_\perp$:

$$H = J_1^{\text{eff}}\sum_{\langle i,j\rangle}\mathbf{S}_i\cdot\mathbf{S}_j + J_2^{\text{eff}}\sum_{\langle i,i'\rangle}\mathbf{S}_i\cdot\mathbf{S}_{i'} + J_\perp\sum_{\langle i,j'\rangle}\mathbf{S}_i\cdot\mathbf{S}_{j'} \tag{6}$$

where $\langle i, j'\rangle$ denotes pairs of out-of-plane nearest neighbors. In the SECNO OP sample, the out-of-plane exchange coupling is estimated to be $J_\perp \approx 6.0 \pm 2.3$ meV, corresponding to a ratio of $J_\perp/J_1 \approx 14\%$ (Supplementary Fig. 9). This ratio is significantly larger than the typical values observed in IL cuprates (∼8%), indicating a distinct degree of three-dimensionality in the magnetic interactions of IL nickelates. It should be noted that, due to the lack of experimental data at momentum positions close to $(0, 0)$, a more detailed characterization of $J_\perp$ lies beyond the scope of this study.

Supplementary Table 1. Linear spin-wave fitting parameters

Sample	$J^{\text{eff}}_{1,\omega_0}$ [meV]	$J^{\text{eff}}_{2,\omega_0}$ [meV]	$J^{\text{eff}}_{1,\omega_{\text{max}}}$ [meV]	$J^{\text{eff}}_{2,\omega_{\text{max}}}$ [meV]
SECNO OP	47.1 ± 4.7	-2.4 ± 3.6	31.6 ± 4.2	-4.5 ± 3.3
PSNO OP	59.2 ± 7.6	-1.6 ± 5.6	38.4 ± 4.2	-7.3 ± 3.3
$Nd_{0.775}Sr_{0.225}NiO_2$ [2]	61.6 ± 4.3	1.0 ± 3.1	–	–

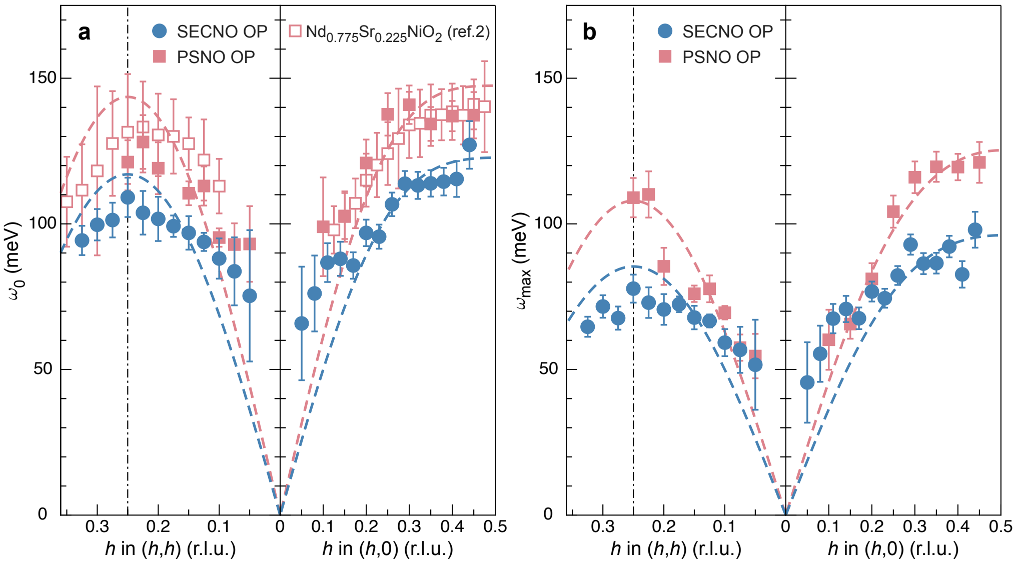

Supplementary Fig. 7. Dispersion relations of the paramagnon (a) pole energy (ω_0) and (b) peak maximum ($\omega_{\max}$) for optimally doped SECNO (blue) and PSNO (filled red). Dashed lines represent fits to the linear spin-wave model for SECNO and PSNO. Paramagnon dispersion for $Nd_{0.775}Sr_{0.225}NiO_2$ (open square), adapted from Ref. [2], is overlaid in (a).

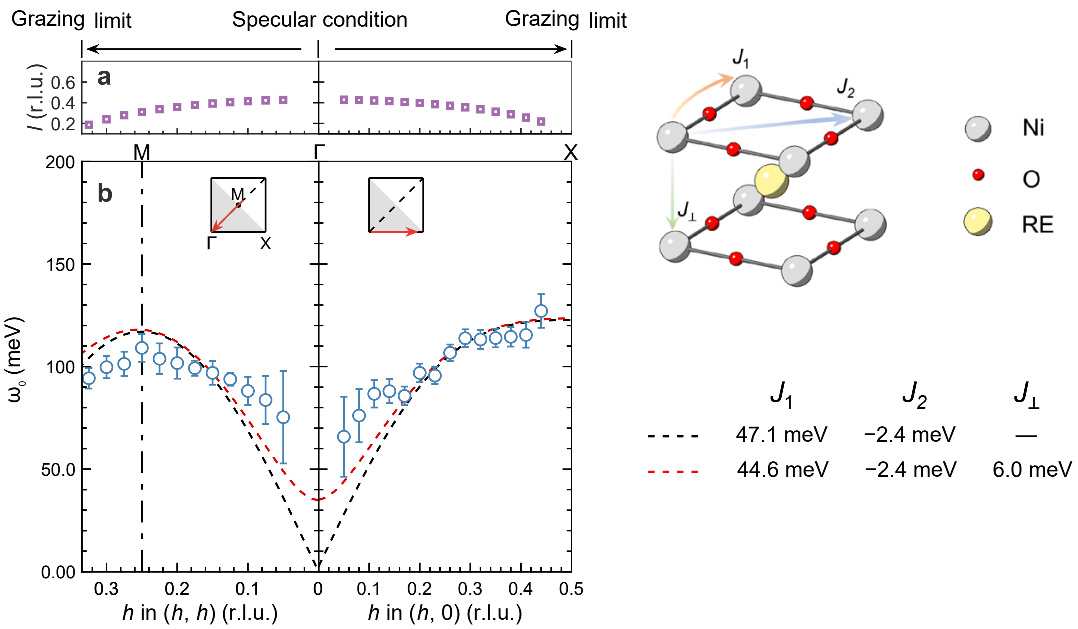

Supplementary Fig. 8. Linear spin-wave fits for SECNO. (a) Out-of-plane momentum l corresponding to each measured spectrum. The relation between the in-plane momentum transfer and the scattering geometry is indicated above the panel. The grazing limit corresponds to the minimum accessible angle between the outgoing/incident beam and sample surface, which is about 10° in our measurements. (b) Linear spin-wave modeling of the paramagnon dispersion for SECNO with (red dashed line) and without (black dashed line) a finite $J_\perp$.

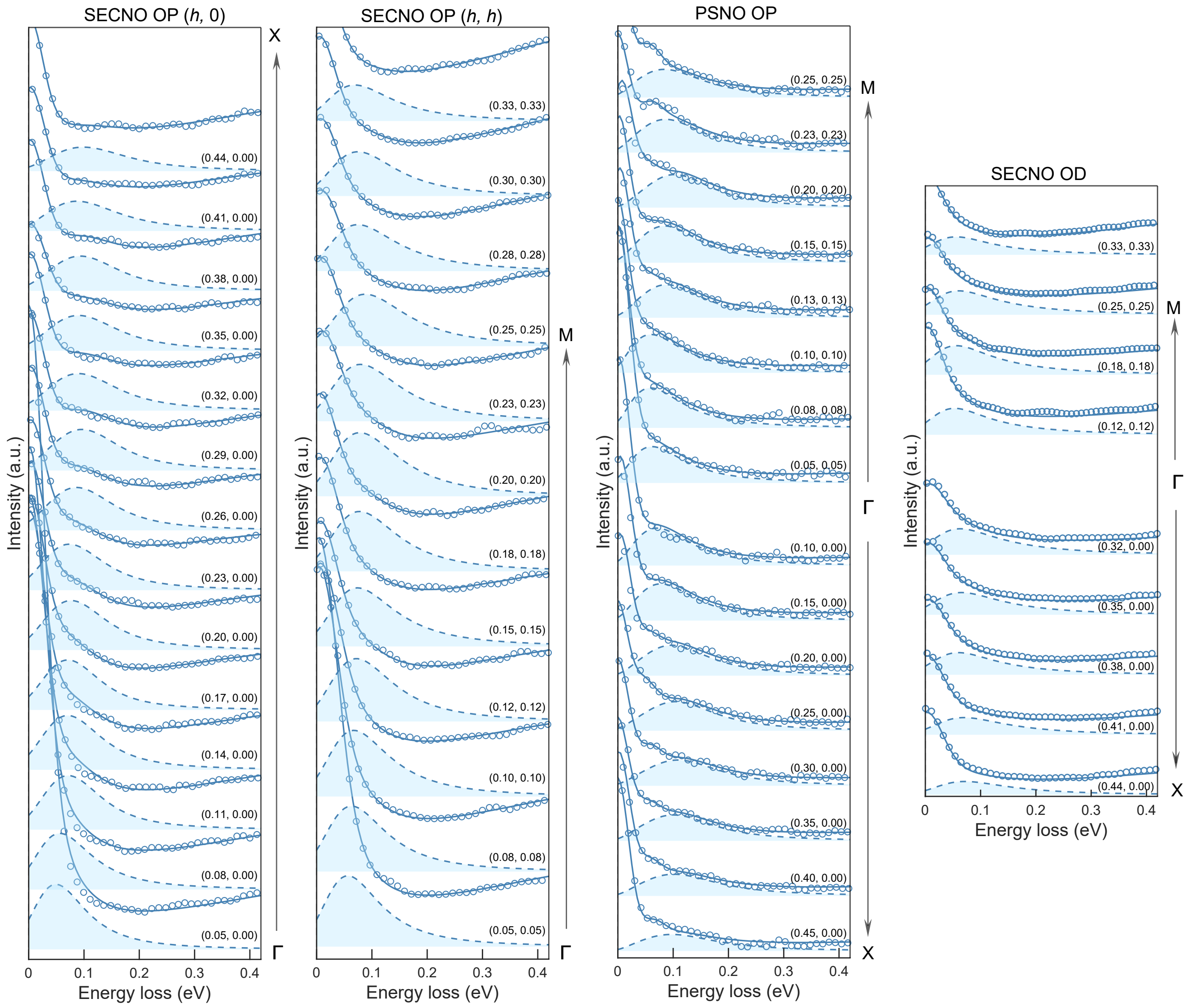

Supplementary Fig. 9. RIXS spectra measured at $2\theta = 150^\circ$ and corresponding fits. Stacked RIXS spectra for SECNO (OP and OD) and PSNO. Open circles represent the raw RIXS data, solid lines denote the total fit, and the light-blue shaded areas indicate the extracted paramagnon component. All spectra are vertically offset for clarity. The corresponding experimental geometry and momentum-transfer values for each spectrum are summarized in Supplementary Table 2.

Supplementary Table 2. Scattering geometry and corresponding momentum transfer

*SECNO OP

Wave vector	α (deg)	h (r.l.u.)	k (r.l.u.)	l (r.l.u.)
$(h, 0, l)$ $(\phi = 0°)$	80.6	0.05	0.00	0.43
$2\theta = 150°$	84.0	0.08	0.00	0.43
	87.4	0.11	0.00	0.42
	91.0	0.14	0.00	0.42
	94.5	0.17	0.00	0.41
	98.3	0.20	0.00	0.40
	101.8	0.23	0.00	0.39
	105.6	0.26	0.00	0.37
	109.7	0.29	0.00	0.36
	113.9	0.32	0.00	0.34
	118.3	0.35	0.00	0.31
	123.2	0.38	0.00	0.29
	128.5	0.41	0.00	0.26
	134.6	0.44	0.00	0.22
(h, h, l) $(\phi = 45°)$	83.0	0.05	0.05	0.43
$2\theta = 150°$	87.0	0.08	0.08	0.42
	91.2	0.10	0.10	0.41
	95.3	0.12	0.12	0.41
	99.6	0.15	0.15	0.39
	104.0	0.18	0.18	0.38
	108.7	0.20	0.20	0.36
	113.6	0.23	0.23	0.34
	118.9	0.25	0.25	0.31
	124.7	0.28	0.28	0.28
	131.5	0.30	0.30	0.24
	139.3	0.33	0.33	0.19
(h, h, l) $(\phi = 45°)$	73.8	0.12	0.12	0.28
$2\theta = 90°$	83.5	0.18	0.18	0.24

*PSNO OP

Wave vector	α (deg)	h (r.l.u.)	k (r.l.u.)	l (r.l.u.)
$(h, 0, l)$ $(\phi = 0°)$	88.1	0.10	0.00	0.44
$2\theta = 154°$	93.7	0.15	0.00	0.43
	99.6	0.20	0.00	0.42
	105.6	0.25	0.00	0.40
	112.1	0.30	0.00	0.37
	119.1	0.35	0.00	0.33
	127.0	0.40	0.00	0.29
	136.6	0.45	0.00	0.23
(h, h, l) $(\phi = 45°)$	84.8	0.05	0.05	0.45
$2\theta = 154°$	88.7	0.08	0.08	0.44
	92.7	0.10	0.10	0.43
	96.8	0.13	0.13	0.42
	101.0	0.15	0.15	0.41
	109.8	0.20	0.20	0.38
	114.6	0.23	0.23	0.36
	119.6	0.25	0.25	0.33

*SECNO OD

Wave vector	α (deg)	h (r.l.u.)	k (r.l.u.)	l (r.l.u.)
$(h, 0, l)$ $(\phi = 0°)$	113.9	0.32	0.00	0.34
$2\theta = 150°$	118.3	0.35	0.00	0.31
	123.2	0.38	0.00	0.29
	128.5	0.41	0.00	0.26
	134.6	0.44	0.00	0.22
(h, h, l) $(\phi = 45°)$	95.3	0.12	0.12	0.41
$2\theta = 150°$	104.0	0.18	0.18	0.38
	118.9	0.25	0.25	0.31
	139.3	0.33	0.33	0.19

[1] M. Rossi, H. Lu, A. Nag, D. Li, M. Osada, K. Lee, B. Y. Wang, S. Agrestini, M. Garcia-Fernandez, J. J. Kas, Y.-D. Chuang, Z. X. Shen, H. Y. Hwang, B. Moritz, K.-J. Zhou, T. P. Devereaux, and W. S. Lee, Phys. Rev. B **104**, L220505 (2021).

[2] H. Lu, M. Rossi, A. Nag, M. Osada, D. F. Li, K. Lee, B. Y. Wang, M. Garcia-Fernandez, S. Agrestini, Z. X. Shen, E. M. Been, B. Moritz, T. P. Devereaux, J. Zaanen, H. Y. Hwang, K.-J. Zhou, and W. S. Lee, Science **373**, 213 (2021).

[3] B. H. Goodge, D. Li, K. Lee, M. Osada, B. Y. Wang, G. A. Sawatzky, H. Y. Hwang, and L. F. Kourkoutis, Proc. Natl. Acad. Sci. U.S.A. **118**, e2007683118 (2021).

[4] H. C. Robarts, M. Barthélemy, K. Kummer, M. García-Fernández, J. Li, A. Nag, A. C. Walters, K. J. Zhou, and S. M. Hayden, Phys. Rev. B **100**, 214510 (2019).

[5] L. Wang, G. He, Z. Yang, M. Garcia-Fernandez, A. Nag, K. Zhou, M. Minola, M. L. Tacon, B. Keimer, Y. Peng, and

Y. Li, Nat. Commun. **13**, 3163 (2022).

[6] F. Rosa, L. Martinelli, G. Krieger, L. Braicovich, N. B. Brookes, G. Merzoni, M. Moretti Sala, F. Yakhou-Harris, R. Arpaia, D. Preziosi, M. Salluzzo, M. Fidrysiak, and G. Ghiringhelli, Phys. Rev. B **110**, 224431 (2024).

[7] F. Rosa, H. Sahib, G. Merzoni, L. Martinelli, R. Arpaia, N. B. Brookes, D. D. Castro, M. Zinouyeva, M. Salluzzo, D. Preziosi, and G. Ghiringhelli, arXiv:2511.02448 (2025).

[8] S. Fan, H. LaBollita, Q. Gao, N. Khan, Y. Gu, T. Kim, J. Li, V. Bhartiya, Y. Li, W. Sun, J. Yang, S. Yan, A. Barbour, X. Zhou, A. Cano, F. Bernardini, Y. Nie, Z. Zhu, V. Bisogni, C. Mazzoli, A. S. Botana, and J. Pelliciari, Phys. Rev. Lett. **133**, 206501 (2024).

[9] L. J. P. Ament, M. van Veenendaal, T. P. Devereaux, J. P. Hill, and J. van den Brink, Rev. Mod. Phys. **83**, 705 (2011).

[10] W. Nolting, Zeitschrift für Physik A Hadrons and Nuclei **255**, 25 (1972).

[11] Q. Gao, S. Fan, Q. Wang, J. Li, X. Ren, I. Biało, A. Drewanowski, P. Rothenbühler, J. Choi, R. Sutarto, Y. Wang, T. Xiang, J. Hu, K.-J. Zhou, V. Bisogni, R. Comin, J. Chang, J. Pelliciari, X. J. Zhou, and Z. Zhu, Nat. Commun. **15**, 5576 (2024).

[12] R. Coldea, S. M. Hayden, G. Aeppli, T. G. Perring, C. D. Frost, T. E. Mason, S.-W. Cheong, and Z. Fisk, Phys. Rev. Lett. **86**, 5377 (2001).
