# Supplementary material for: Persistent paramagnons in high-temperature infinite-layer nickelate superconductors
Source: Nat Commun. 2026 May 20;17:6645. doi: 10.1038/s41467-026-73083-3 (PMC13381760; doi:10.1038/s41467-026-73083-3)
Supplement: Supplementary file 2 — Transparent Peer Review file [file 41467_2026_73083_MOESM2_ESM.pdf]

nature portfolio

Peer Review File

Persistent paramagnons in high-temperature infinite-layer nickelate superconductors

Corresponding Author: Professor Qisi Wang

This file contains all reviewer reports in order by version, followed by all author rebuttals in order by version.

Version 0:

Reviewer comments:

Reviewer #1

(Remarks to the Author)
The authors have studied (Sm,Eu,Ca,Sr)NiO2 infinite-layer nickelate superconducting thin films using Ni L-edge resonant inelastic x-ray scattering (RIXS). They measured optimally doped and overdoped samples and compared their data with Pr-nickelate films that have a lower T_c than Sm-nickelate. Overall, RIXS experiments and data analyses were done well. However, there are a few concerns, mostly due to the nature of the limited experimental tool (not due to the authors' capabilities):

1. The main observation is the softening of paramagnon dispersion in Sm-nickelate compared to Pr-nickelate, although T_c is 2 times higher in Sm-nickelate. From the Heisenberg spin Hamiltonian model fit, the exchange interaction is estimated to be about 20% reduced in Sm-nickelates. However, the softening at the zone boundary seems to be only around 20 meV. Considering the energy resolution (67 meV or 46 meV) of soft x-ray RIXS, it raises some concerns, although I believe that the authors have carried out the experiment carefully and subtracted the strong elastic signal consistently.

2. Two different samples were measured at two different beamlines (Taiwan and England), respectively, and their data are compared in this manuscript. Ideally, it shouldn't affect the data. However, we are not living in an ideal world, and under such poor energy resolution of RIXS, it raises some concerns. For example, by looking at the contour color maps of Fig. 2d and 2e, clear differences in momentum and energy resolutions make it hard to be convinced. If the authors have measured them at the same beamline under identical conditions, it would enhance the credibility of the data.

3. Looking at Fig. 3 and Supple. Fig. 2, the energy doesn't seem to go to zero as approaching (0,0). Is there a paramagnon gap at the zone center, presumably due to anisotropy or something else?

4. I understand that the limited energy resolution of soft x-ray RIXS is a challenging issue for now. However, how about double-checking with Raman scattering experiments? Although it doesn't provide momentum information, the authors may observe magnon gap energies or bi-magnon scattering, from which they can obtain the exchange interaction value. The energy resolution of Raman scattering is much better than RIXS.

5. The samples were measured only at 30 K and 16 K for Sm- and Pr-nickelate, respectively. I guess that the authors tried to measure them near T_c. However, is there no temperature dependence?

6. The discussion part is somewhat hand-waving and weak. Although this manuscript is focused on experimental results, it would be great to include more detailed mechanisms, insights, or theoretical calculations if possible.

Reviewer #2

(Remarks to the Author)
In the manuscript “Persistent paramagnons in high-temperature infinite-layer nickelate superconductors”, by Y. Yan at al., the authors study magnetic excitations in hole-doped infinite-layer nickelate thin films. They measure resonant inelastic x-ray

scattering spectra for different samples and scattering geometries and fit the magnetic part of their spectral functions using damped harmonic oscillator model framework. The magnetic excitations are assigned as paramagnons. From the obtained dispersion relation across the first Brillouin zone in energy and momentum domain, they extract magnetic exchange coupling between the nearest and next-nearest spins, using the Heisenberg spin-chain model. Results from superconducting, optimally-doped Sm-based film are compared with results from overdoped Sm-based sample and superconducting, optimally-doped Pr-based sample. In addition, the exchange coupling is extracted for an optimally doped Nd-based film, using literature data from H. Lu et al., Science 373, 213 (2021) (presented in the Supplementary Information).

The main claim of the work is the observation of a decreased magnetic exchange coupling for infinite-layer nickelate systems with higher superconducting transition temperature, Tc (here represented by $Sm_{0.73}Eu_{0.2}Ca_{0.07}NiO_2$), compared to compounds with lower Tc (represented by $Pr_{0.8}Sr_{0.2}NiO_2$). This behavior would be indeed intriguing, since it would contrast the reported behavior for cuprate superconductors. The Sm-based system is itself very interesting, due to its particularly high superconducting transition temperature of 30 K.

Unfortunately, the presented data and the discussion on the results is not complete, limiting the possibility to judge on data quality and on the robustness of findings. Therefore, in the current state I cannot give a positive assessment about the quality of this work, as major improvements to the manuscript are still required.

In particular, apart from Fig. 2a,b, displaying small fraction of measured line spectra, raw data is not presented. All other figures (including the Supplementary Information) show data, where large fraction of the spectral weight was already subtracted. This is not very transparent and creates doubts about the data set. Both, as reviewer and reader I would expect to be able to view the measured line spectra, as well as the fitted spectra, including decomposition into single contributions. This can be part of Supplementary Information, similarly as it is done for many references cited in this work.

The energy axis in Fig. 2d,e does not seem to be consistent with the energy scales of other presented figures (in Fig. 2d.e magnetic excitations appear at much lower energy loss than in other graphs). Therefore, the authors should check their graphs carefully. Also, there are two terms used in Fig. 2c and 2d, e without being specified: “position of maximum intensity” and “peak energy” (on top of different energy scales between these figures). These terms should be defined in the Manuscript. Currently, the presented data does not look consistent to me.

I am also missing discussion on possible phonon contribution to the observed magnetic excitations. The position of the magnetic excitations is low in energy, merely slightly above the instrumental energy resolution and the excitations are also broad. Previous work reported on phonons in Ni L3 RIXS from infinite-layer nickelates (see e.g. H. Lu et al. and F. Rosa et al., PRB 110, 224431 (2024)). In the publication of Rosa et al. phonon excitations merge with magnetic excitations upon hole doping. Since there no raw data is shown in this work, it is puzzling to the reader whether phonons were visible in the spectra. In any case, the question how the presence of phonons might influence the finding of this study should be addressed.

I noticed that not all data points presented in Fig. 2 are included in Fig. 3e. It seems that all values below q=0.1 rlu are missing. There is probably a good reason for not considering this part of data, however the authors should justify their decision in the manuscript in order to be transparent.

Another major point is that the presentation of the fitting results from the damped harmonic oscillator model is not complete. I am in particularly missing information on the susceptibility X’ and results on the damping constant γ. I would expect to see discussion of size of γ and comparison to findings from other infinite-layer nickelate compounds (see references in the Manuscript), which would be a critical part for discussion of robustness of the results. This data could be included in the Supplemental Information.

Indications for softening of magnetic excitations in infinite-layer nickelates was reported already in previous studies and therefore the authors should cite this work when discussing their own results in Lines 175-182 and 206-213 (see H. Lu et al. and F. Rosa et al., mentioned above). At the same time, contrary to the manuscript, this topic is still controversial for the cuprate systems. Besides reports of softening of magnetic excitations with doping, there are publications claiming opposite behavior (see Y. Y. Peng, Phys. Rev. B 98, 144507 (2018) and H. C. Robarts, Phys. Rev. B 100, 214510 (2019)).

Further remarks, where described effects could be better specified/ quantified:
- Lines 140-142: no detailed comparison between crystal field in Sm-based and Nd-based compounds is presented, therefore it cannot be claimed that the crystal field environment is similar. However, the authors might state that the dd excitations don’t show obvious differences.
- Lines 148-149: how does the resonant behavior demonstrate the electronic origin? (what would be an alternative origin?)
- Lines 163-166: increasing energy with increasing momentum is not sufficient to claim characteristic dispersion of square-lattice antiferromagnetic paramagnons, but rather shows an agreement.
- Lines 171-172: how broad is the width of paramagnons?
- Lines 172-174: from the presented data I do not see that the spectral weight is suppressed and the linewidth broadens (with increasing momentum). In case this finding is important, the authors should provide a graph showing these effects and quantify them.

Remarks related to figures, partially due to missing information:
- At which momentum transfer was data in Fig. 1 it measured? Alternatively, a table/graph relating the incidence angle with momentum transfer could be provided in the Supplementary Information.

- At which photon energy were spectra presented in Fig. 2 and 3 obtained?
- At which momentum transfer was each line plot in Fig. 2c measured?
- The color-scale in Fig. 2 d,3 is not favorable, since there is little contrast between yellow, white and blue.
- More horizontal tics for all graphs in Fig. 3 would be useful to follow the data.
- Indicating size of energy resolution as horizontal line either in the false-color plot or in the dispersion graphs would be informative, as it presents a limitation to the sensitivity of the data

Reviewer #3

(Remarks to the Author)
Yan and coworkers are reporting a RIXS study on the magnetic excitations of some nickelate superconductors. In particular, the authors find that paramagnons persist in optimally doped (OP) and even overdoped (OD) Sm-based infinite-layer (IL) nickelate SCs with a Tc up to 40K. Most importantly, they report that the peak energy of the magnons of the OP sample with Tc of 40K is lower than for corresponding Pr-based IL-nickelates SCs with a lower Tc of only 10K. This suggests a negative correlation between Tc and the in-plane exchange interaction J that is different from the cuprates and therefore of interest for researchers from within the high Tc community. Their suggestions that multi-band effects due to hybridization between rare earth 5d and Ni 3d orbitals levels and/or Kondo-correlations due to enhanced c-axis coupling may need to be considered, will certainly motivate further experimental and theoretical work along these lines. However, I am not quite sure whether their results and conclusions will be of sufficient interest for researchers beyond the high Tc community. I am therefore reluctant to propose this manuscript for a journal like Nature Communications.

Moreover, I have some major questions regarding the presentation and interpretation of the data.
Most importantly, there appears to be a large offset in the energy of the peak intensity in the maps of Fig. 2d-e and the magnon peak energies shown in Fig. 2c and Fig. 3. I wonder what causes this large difference in peak energies by almost a factor of two? Is it due to a mistake in labeling the y-scale in Figs. 2d and 2e? Otherwise, how can it be that that the intensities peak at such difference energies in Fig. 2c and Fig. 2d?
The authors mention that a possible explanation for their finding, that Tc does not scale with the in-plane J, is the hybridization with the rare earth ions and a subsequent enhanced 3D character of the Sm based system. This raises the question how this would affect their analysis of the RIXS data where it seems to me that the out-of-plane component of the scattering vector has not been considered?
Another technical question. The SECS and PECS samples have been measured at different instrumental setups with different energy resolutions. This makes me wonder how the authors can be sure that the lower energy resolution of the SECS data does not lead to an apparent reduction of the fitted peak energy?
A related question. In the technical part on the RIXS experiments the authors state that “The beamline-specific photon energy offset has not been considered”. I do not understand what exactly this means. Have they shifted the energy scales to match the positions of the elastic peaks? Otherwise, how can they be sure that the shift of the magnon peak energy is not caused by different energy offsets of the spectrometers?

Minor comments.
In the cuprates the rare earth can also matter, i.e. see Pr doped YBCO for which Tc and metallicity is suppressed.
I suggest that the references concerning the positive correlation between Tc and J in the cuprates should be given already in the introduction, i.e. not only at the final discussion.
Finally, I find the abbreviations SECS and PSNO somewhat inconsistent, i.e., why is the oxygen (O) included for the latter but not the former system?

Version 1:

Reviewer comments:

Reviewer #1

(Remarks to the Author)
The authors reasonably addressed my questions, as well as those of the other reviewers, and revised both the manuscript and the supplementary material.

Reviewer #2

(Remarks to the Author)
I would like to thank the authors for addressing my comments. The authors now show more details on the measured spectra in the supplement. Nevertheless, I believe that the new version of the manuscript still needs improvements:

1) I would recommend that the authors display an overview with all analyzed RIXS line spectra in the Supplementary information (as done e.g. in Ref. [7]).
2) Comparing Fig. R7 a) and b) I don’t see a reason why the data points close to the Gamma point should not be shown in the Manuscript. They have larger error bars than the data points for larger momentum transfer, however the fitting results are in both cases similar and within the error bars. Therefore, I would recommend to replace Fig. 3 e) with Fig. R7 a) (without the blue shaded area), which includes all data points.

3) Lines 169-182: this section is not written in a very coherent way. On one side the authors still discuss the origin of the observed 0.1 eV excitation and at the same time compare already details of their results with other systems. The two should be separated. Also, the discussion on possible non-isotropy of magnetic excitations in SECNO can be ideally moved to later section, where modelling of the effective exchange interactions is discussed.
4) Lines 183-193: I find the statement of a general trend too strong. The authors should rather say that two samples with different hole doping were studied and that the energy of magnetic excitations of the sample with higher doping is very likely lower (this is what can be seen from Fig. 3.). Later, in the discussion part, comparison to other nickelate systems and cuprates shall be made, where it can be argued that the presented observations from SECNO points toward similar trends. In fact, I am a puzzled when comparing Fig. 3e with Fig. S1, since the differences in the two displayed RIXS spectra in the later seem to be much larger than the results presented in Fig. 3e. What explains this deviation? Besides the two graphs, there is no other data presented for the overdoped sample, neither complete analysis results.
5) Lines 217-219: In my opinion this sentence can be moved to the results section. What are “extended defects” and why are the samples free of such defects?
6) Lines 219-224: Can be merged with discussion of possible softening of magnetic excitations with doping for SECNO. However, in my opinion this paragraph does not fit well in between the previous and the following paragraph and should be moved somewhere else.
7) Lines 275-277: which particular differences are meant here?
8) Lines 277-281: what are the alternative design principles for superconducting nickelates and cuprates?

Reviewer #3

(Remarks to the Author)
The authors have responded well to the questions and suggestions that I had raised in my previous report. In particular, they have answered well to all my technical questions/reservations. Moreover, they have provided rather a convincing discussion of why their results could be of interest for a broader research community, and not just the experts in the particular field of high-Tc superconductors.
I am therefore inclined to agree with the authors that this manuscript is suitable for publication in Nature Communications.

Version 2:

Reviewer comments:

Reviewer #2

(Remarks to the Author)
The authors have addressed all my questions. I have no further comments.

Open Access This Peer Review File is licensed under a Creative Commons Attribution 4.0 International License, which permits use, sharing, adaptation, distribution and reproduction in any medium or format, as long as you give appropriate

credit to the original author(s) and the source, provide a link to the Creative Commons license, and indicate if changes were made.
In cases where reviewers are anonymous, credit should be given to 'Anonymous Referee' and the source.
The images or other third party material in this Peer Review File are included in the article's Creative Commons license, unless indicated otherwise in a credit line to the material. If material is not included in the article's Creative Commons license and your intended use is not permitted by statutory regulation or exceeds the permitted use, you will need to obtain permission directly from the copyright holder.
To view a copy of this license, visit https://creativecommons.org/licenses/by/4.0/

Reviewer #1 (Remarks to the Author):

The authors have studied (Sm,Eu,Ca,Sr)NiO2 infinite-layer nickelate superconducting thin films using Ni L-edge resonant inelastic x-ray scattering (RIXS). They measured optimally doped and overdoped samples and compared their data with Pr-nickelate films that have a lower T_c than Sm-nickelate. Overall, RIXS experiments and data analyses were done well. However, there are a few concerns, mostly due to the nature of the limited experimental tool (not due to the authors' capabilities):

Authors: We thank the referee for the precise summary of our study and for the encouraging comments regarding the quality of our experiments and data analysis. In the following, we respond to each point and discuss why the conclusions of this study are not limited by the experimental method. For consistency, we have now changed the abbreviation of the Sm-system into SECNO throughout this reply and the revised manuscript.

1. The main observation is the softening of paramagnon dispersion in Sm-nickelate compared to Pr-nickelate, although T_c is 2 times higher in Sm-nickelate. From the Heisenberg spin Hamiltonian model fit, the exchange interaction is estimated to be about 20% reduced in Sm-nickelates. However, the softening at the zone boundary seems to be only around 20 meV. Considering the energy resolution (67 meV or 46 meV) of soft x-ray RIXS, it raises some concerns, although I believe that the authors have carried out the experiment carefully and subtracted the strong elastic signal consistently.

Authors: We thank the referee again for recognizing the quality of our measurements. The referee is raising an important point. Technically, while the instrumental energy resolution contributes to the overall spectral broadening, the centroid of a peak can often be determined with a precision significantly better than the instrumental resolution, given sufficient statistics and an appropriate fitting model. The high statistics of our data allows us to determine the peak positions with uncertainty well within the energy resolution, as indicated by the error bars in the dispersion plots.

In our analysis, we applied a fitting model commonly employed in RIXS studies to describe low-energy excitations involving paramagnons [e.g. nickelates: H. Lu et al., Science 373, 213 (2021); cuprates: H. Robarts et al., PRB 100, 214510 (2019); iron-selenide: X. Lu et al., Nat. Phys. 18, 806, (2022)]. As shown in Suppl. Fig. 7a (Fig. R4), applying the same analysis approach, we obtain a dispersion for the Pr-based sample comparable with the Nd-based sample reported previously [H. Lu et al., Science 373, 213 (2021)], while the paramagnon energy for the Sm-based samples exhibits a systematic reduction across the entire Brillouin zone. This cross-material comparison, under matched analysis conditions, supports that the observed ~20 meV zone-boundary softening in Sm-nickelate is intrinsic rather than an artifact of resolution or fitting.

Moreover, we stress that this conclusion is consistent across data obtained with different scattering geometries (see Suppl. Fig. 5 and Fig. R3), and is robust whether or not including phonon contributions (see Suppl. Note 2B and Fig. R5). We have also conducted additional spectral moment analysis to confirm the reliability of the fitting results (see Suppl. Note 2D and Fig. R8). Taken together, the convergence of results across geometries, modeling choices, and moment analysis provides strong evidence that the softening of the paramagnon dispersion in Sm-nickelate relative to Pr-nickelate is a genuine, sample-intrinsic effect.

Corresponding revisions: To demonstrate the robustness and validity of our conclusion on the reduced magnon energy in Sm-based system, we have added new analyses in Suppl. Notes 2B,2C,2D, which include Suppl. Figs. 2-6.

2. Two different samples were measured at two different beamlines (Taiwan and England), respectively, and their data are compared in this manuscript. Ideally, it shouldn't affect the data.

However, we are not living in an ideal world, and under such poor energy resolution of RIXS, it raises some concerns. For example, by looking at the contour color maps of Fig. 2d and 2e, clear differences in momentum and energy resolutions make it hard to be convinced. If the authors have measured them at the same beamline under identical conditions, it would enhance the credibility of the data.

Authors: As correctly noted by the referee, the comparison between measurements from two beamlines should not affect the results, provided that both experiments were conducted with sufficient energy resolution. In principle, as long as the intrinsic linewidth of the feature exceeds the instrumental resolution, the excitation can be reliably resolved.

To illustrate this point, Fig. R1 presents a comparison between the lifetime parameter 2γ, which describes the intrinsic energy width for our PSNO (pink) and SECNO (blue) samples, and the corresponding experimental energy resolutions (dashed lines). The paramagnon linewidths are significantly broader than the instrumental resolutions, and are also comparable to those reported for other infinite-layer nickelates around optimal doping [H. Lu et al., Science 373, 213 (2021), S. Fan et al, PRL 133, 206501 (2024)]. This confirms that the observed energy broadening is intrinsic in origin, rather than being limited by the resolution.

Furthermore, although the PSNO data were collected with slightly better resolution, the energy widths for paramagnons in PSNO and SECNO are comparable. This suggests that the small difference in resolution between the two beamlines is negligible relative to the intrinsic broadening of the paramagnons, and thus does not influence the reliability of our results or analysis.

Regarding the momentum resolution, the referee's comment serves as a valuable reminder. Notably, the momentum resolution across the two beamlines is comparably high. At Ni L-edge, the momentum resolution is approximately ±0.01 $Å^{-1}$, which corresponds to ±0.006 r.l.u with a lattice constant of a=3.86Å [J. Synchrotron Rad. 29, 563 (2022), Nat. Mater. 21, 1116 (2022)]. This resolution is well below the intrinsic momentum width of the paramagnons.

Corresponding revisions: In line with the referee's comment, we have added the information of the momentum resolution and discussion on the energy resolution to the Methods section of the revised manuscript.

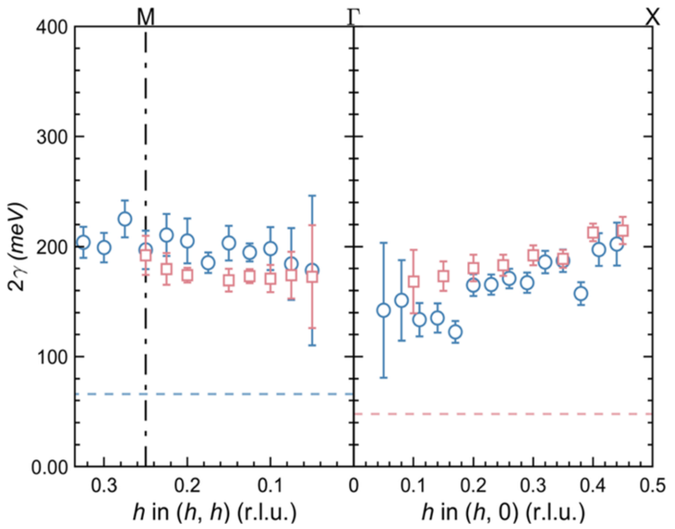

Fig. R1. Paramagnon energy width (2γ) extracted from the DHO fitting for optimally doped SECNO (blue) and PSNO (red), the blue (red) dash line represents the energy resolution (FWHM) of 41A1 (I21) beamline, the paramagnon energy width is significantly broader than both of the resolution.

3. Looking at Fig. 3 and Suppl. Fig. 2, the energy doesn't seem to go to zero as approaching (0,0). Is there a paramagnon gap at the zone center, presumably due to anisotropy or something else?

Authors: The referee is raising an important point. A spin gap can open at the zone center due to anisotropy or the presence of a finite out-of-plane exchange coupling $J_\perp$. Although small, the latter has been considered in some RIXS studies of undoped cuprates [e.g. Peng et al., Nat. Phys. **13**, 1201, (2017)]. If the system exhibits a more pronounced three-dimensional magnetic character, the associated spin-gap energy may increase accordingly.

In Fig. R2, we compare the paramagnon dispersions obtained with and without including a finite $J_\perp$ in the model. This demonstrates that the inclusion of $J_\perp$ has marginal influence on the fitted value of the in-plane exchange coupling $J_{//}$, which primarily governs the paramagnon zone-boundary energy. Therefore, our key conclusions will remain unchanged irrespective of the presence of a spin gap.

Corresponding revisions: We have added the above analysis and discussions in Suppl. Note 3, and included Fig. R2 as Suppl. Fig. 8.

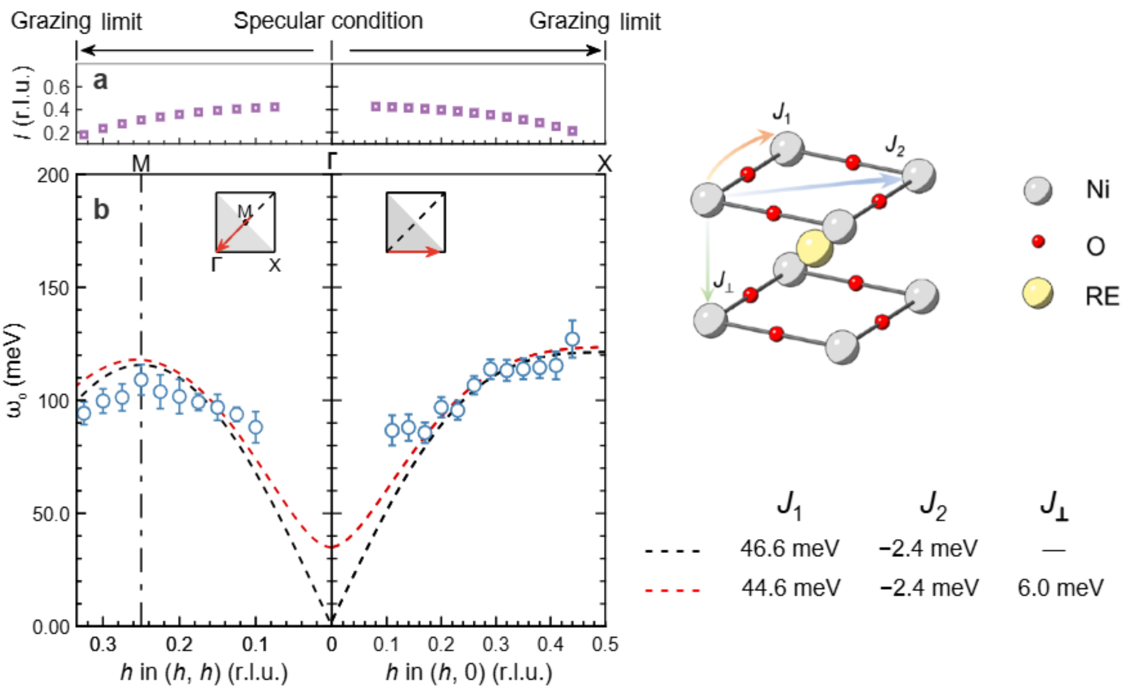

Fig. R2. Spin-wave fits with and without an out-of-plane coupling for SECNO. (a) Out-of-plane momentum component *L* corresponding to each measured spectrum. The relation between the in-plane momentum transfer and the scattering geometry is indicated above the panel. The grazing limit corresponds to the minimum angle between the outgoing/incident beam and sample surface, which is about 10° in our measurements. (b) Linear spin-wave fits of the paramagnon dispersion for SECNO with (red dashed line) and without (black dashed line) a finite $J_\perp$.

4. I understand that the limited energy resolution of soft x-ray RIXS is a challenging issue for now. However, how about double-checking with Raman scattering experiments? Although it doesn't provide momentum information, the authors may observe magnon gap energies or bi-magnon

scattering, from which they can obtain the exchange interaction value. The energy resolution of Raman scattering is much better than RIXS.

Authors: We thank the referee for this insightful suggestion and agree that Raman scattering provides excellent energy resolution and can indeed probe magnetic excitations through bi-magnon processes.

As the referee points out, Raman can probe bi-magnon excitations at the zone center. In the limit of negligible magnon-magnon interaction, single- and bi-magnon excitations energies scale by a factor of two. However, this scaling is non-trivial in the limit of where magnon-magnon interactions are non-negligible [see e.g., Q. Wang et al., Nat. Commun. 15, 5348 (2024)]. Like in the cuprates, we consider the nickelates to host significant magnon-magnon interactions. For this reason, the interpretation of Raman experiments is not obvious when it comes to single magnon excitations (even at q = 0).

5. The samples were measured only at 30 K and 16 K for Sm- and Pr-nickelate, respectively. I guess that the authors tried to measure them near T_c. However, is there no temperature dependence?

Authors: It would be of great interest to investigate the temperature dependence of the low-energy spin excitations across T_c. However, 30 K corresponds to an energy scale of 2.8 meV which is more than ten times smaller than the intrinsic magnon scattering width. For this reason, we do not expect any significant temperature dependence to be resolved upon crossing into the superconducting state.

6. The discussion part is somewhat hand-waving and weak. Although this manuscript is focused on experimental results, it would be great to include more detailed mechanisms, insights, or theoretical calculations if possible.

Authors: We thank the referee for this valuable suggestion. In the revised manuscript, we have expanded the discussion of the mechanisms that may govern the observed modulation of superconductivity and magnetic interactions. In particular, we now highlight prevailing theoretical scenarios alongside recent experimental insights that are closely related to the interpretation of our results. The following discussions has been added to the revised manuscript:

Theoretical perspectives: "*The enhanced three-dimensionality may also strengthen Kondo-like coupling effects, and thereby provide additional pairing strength. In such a Mott-Kondo scenario, the pairing symmetry is predicted to undergo a transition from d to d+is as the Kondo coupling increases, which can be examined experimentally in future. Conversely, a recent polarization-dependent ARPES study suggests that the additional electron Fermi pocket originates from the interstitial s states rather than rare-earth 5d orbitals. The rare-earth element modifies the electronic and magnetic structures through a chemical pressure effect. Although the precise role of rare-earth ions in the electronic structure and superconductivity of IL nickelates remains under investigation, our findings indicate that the reduced size of rare-earth ions effectively tunes the magnetic interactions towards a more three-dimensional structure, which may be the key for the enhanced T_c.*"

New experimental insights: "Moreover, the existence of significantly stronger $J_\perp$ in infinite-layer nickelates compared to that of cuprates has been suggested by a recent RIXS study (see Supplementary Note 3 for more discussions".... "An absence of proportionality between T_c and in-plane exchange coupling has also been recently observed in $PrNiO_2$ grown on different substrates."

With these enhancements, the significance of our findings is further reinforced.

Reviewer #2 (Remarks to the Author):

In the manuscript "Persistent paramagnons in high-temperature infinite-layer nickelate superconductors", by Y. Yan at al., the authors study magnetic excitations in hole-doped infinite-layer nickelate thin films. They measure resonant inelastic x-ray scattering spectra for different samples and scattering geometries and fit the magnetic part of their spectral functions using damped harmonic oscillator model framework. The magnetic excitations are assigned as paramagnons. From the obtained dispersion relation across the first Brillouin zone in energy and momentum domain, they extract magnetic exchange coupling between the nearest and next-nearest spins, using the Heisenberg spin-chain model. Results from superconducting, optimally-doped Sm-based film are compared with results from overdoped Sm-based sample and superconducting, optimally-doped Pr-based sample. In addition, the exchange coupling is extracted for an optimally doped Nd-based film, using literature data from H. Lu et al., Science 373, 213 (2021) (presented in the Supplementary Information).

The main claim of the work is the observation of a decreased magnetic exchange coupling for infinite-layer nickelate systems with higher superconducting transition temperature, Tc (here represented by Sm0.73Eu0.2Ca0.07NiO2), compared to compounds with lower Tc (represented by Pr0.8Sr0.2NiO2). This behavior would be indeed intriguing, since it would contrast the reported behavior for cuprate superconductors. The Sm-based system is itself very interesting, due to its particularly high superconducting transition temperature of 30 K.

Authors: We thank the referee for the concise summary of our experimental methodology and main results. The referee correctly captured both the scope of our study and the key finding regarding the relationship between magnetic exchange coupling and superconducting transition temperature in infinite-layer nickelates.

Unfortunately, the presented data and the discussion on the results is not complete, limiting the possibility to judge on data quality and on the robustness of findings. Therefore, in the current state I cannot give a positive assessment about the quality of this work, as major improvements to the manuscript are still required.

Authors: Following the referee's suggestion, we have expanded the presentation of the data by including raw data in Suppl. Figs. 2,4,5, and conducted additional analysis concerning the possible phonon contributions and spectral moments, which are included as Suppl. Note 2B,2D and Suppl. Figs.2-4,6. These expansions strengthen the data quality and the robustness of our conclusions.

In particular, apart from Fig. 2a,b, displaying small fraction of measured line spectra, raw data is not presented. All other figures (including the Supplementary Information) show data, where large fraction of the spectral weight was already subtracted. This is not very transparent and creates doubts about the data set. Both, as reviewer and reader I would expect to be able to view the measured line spectra, as well as the fitted spectra, including decomposition into single contributions. This can be part of Supplementary Information, similarly as it is done for many references cited in this work.

Authors: Following the referee's suggestion, we have expanded the Supplementary Information to include more raw RIXS spectra, covering both samples, along different high-symmetry directions (see Suppl. Figs. 2, 4 and Fig. R5).

To strengthen the robustness of our analysis, we have also included a comparison of raw spectra measured at different scattering angles (see Fig. R3 and Suppl. Fig. 5). With a scattering angle of

2θ=90°, the elastic scattering is significantly suppressed and the low-energy part becomes dominated by the magnetic mode. The comparison clearly demonstrates that the extracted paramagnon components are consistent across different scattering angles. Since the range of in-plane momentum transfer decreases at a smaller scattering angle, the majority of the data are taken at the maximum scattering angle (150° at 41A and 154° at I21) to map-out the dispersion.

A more detailed discussion of the spectral quality, the raw experimental data, and the fitting results for these spectra is provided below, where we address the referee's concerns point by point.

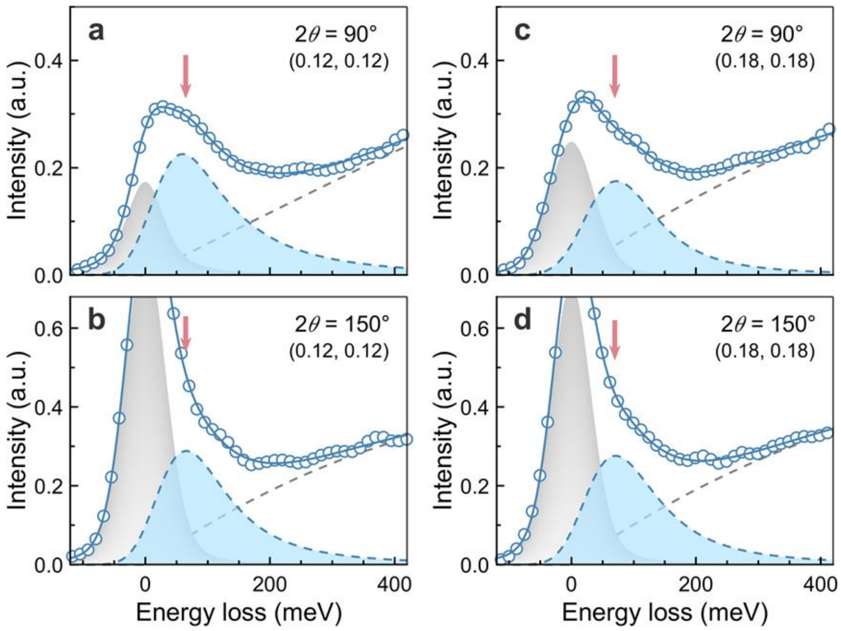

Fig. R3. Comparison of raw RIXS spectra measured at scattering angles for the same in-plane wave vector. (a,b) RIXS spectra at (0.12,0.12) measured with (a) 2θ = 90° and (b) 2θ = 150°. (c,d) RIXS spectra at (0.18,0.18) measured with (c) 2θ = 90° and (d) 2θ = 150°. The shaded areas and dashed lines denote fitting components as described in the manuscript. Red arrows indicate the peak positions of the magnetic components.

The energy axis in Fig. 2d,e does not seem to be consistent with the energy scales of other presented figures (in Fig. 2d.e magnetic excitations appear at much lower energy loss than in other graphs). Therefore, the authors should check their graphs carefully. Also, there are two terms used in Fig. 2c and 2d, e without being specified: "position of maximum intensity" and "peak energy" (on top of different energy scales between these figures). These terms should be defined in the Manuscript. Currently, the presented data does not look consistent to me.

Authors: We thank the referee for the suggestion to clarify the definitions of different parameters. Both terms "*position of maximum intensity*" and "*peak energy*", refer to the position of intensity maximum of the damped harmonic oscillator (DHO) function (ω_{max}), which is lower than the pole energy (ω_0) of the undamped case. A comprehensive discussion on different characteristic energies of a paramagnon can be found in H. Robart et al., PRB 214510 (2019), and J. Lamsal et al., PRB 93, 214513 (2016) — we have now cited these references.

The red arrows in Fig. 2c and white circles in Figs. 2d and 2e indicate ω_{max}. In Fig. 3, the dispersion

of ω_0 is presented. As shown in Suppl. Fig. 7 (Fig. R4), we have analysed dispersions extracted from both characteristic energies ω_0 and ω_{max}, yielding a consistent trend and conclusion — Pr- and Nd-based samples exhibit a higher magnon bandwidth than the Sm-based samples.

To avoid ambiguity, we now define ω_{max} and ω_0 explicitly at an earlier stage of the manuscript and use the terminology consistently throughout the text and figure captions. In addition, as the referee carefully noticed, an error in the energy-axis labeling occurred during the preparation of Figs. 2d and 2e, where the zero-energy position was not properly aligned. This has now been corrected in the revised manuscript. We thank the referee for pointing this out.

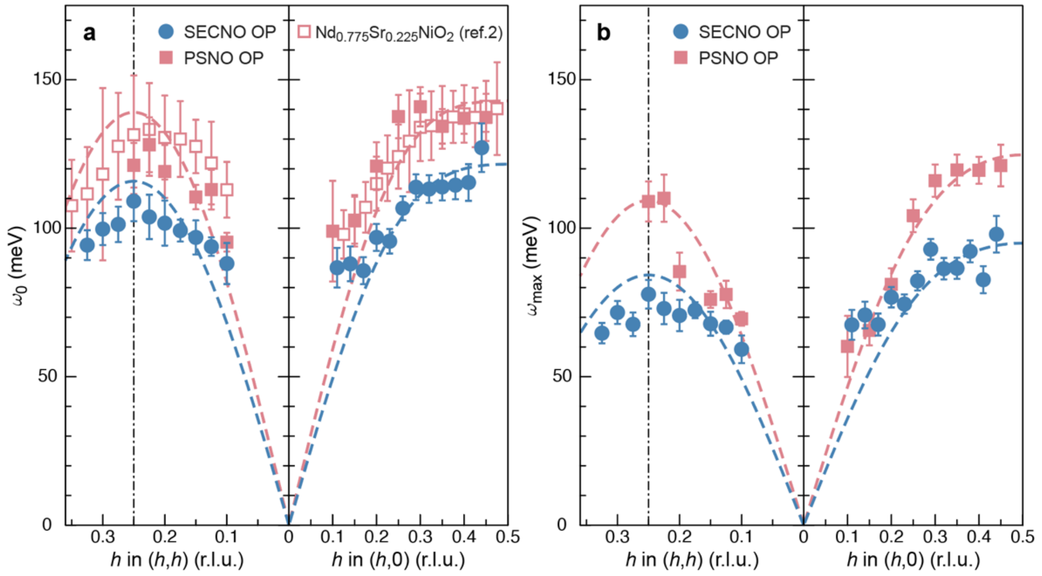

Fig. R4. Dispersion relations of the paramagnon (a) pole energy (ω_0) and (b) peak maximum (ω_{max}) for optimally doped SECNO (blue) and PSNO (filled red). Dashed lines represent fits to the linear spin-wave model for SECNO and PSNO. Paramagnon dispersion for $Nd_{0.775}Sr_{0.225}NiO_2$ (open square), adopted from H. Lu et al., Science 373, 213 (2021), is overlaid in (a).

I am also missing discussion on possible phonon contribution to the observed magnetic excitations. The position of the magnetic excitations is low in energy, merely slightly above the instrumental energy resolution and the excitations are also broad. Previous work reported on phonons in Ni L3 RIXS from infinite-layer nickelates (see e.g. H. Lu et al. and F. Rosa et al., PRB 110, 224431 (2024)). In the publication of Rosa et al. phonon excitations merge with magnetic excitations upon hole doping. Since there no raw data is shown in this work, it is puzzling to the reader whether phonons were visible in the spectra. In any case, the question how the presence of phonons might influence the finding of this study should be addressed.

Authors: As the referee has pointed out, upon doping, the magnetic excitations shift toward lower energies and progressively overlap with the phonon energy scale. To evaluate the influence of possible phonon contributions, we follow the procedure used in H. Lu et al., Science **373**, 213 (2021), and introduce a phonon mode of ~70 meV into our fitting model. In this model, the relative phonon intensity is estimated based on previous RIXS studies of infinite-layer nickelates [e.g. H. Lu et al., Science 373, 213 (2021)], and allowed to vary by ±30% — similar to the treatment in H. Lu et al., Science 373, 213 (2021) for doped samples. In this fashion, we systematically evaluate the

uncertainty associated with the phonon contribution and quantify its potential impact on the extracted magnetic energy scale, linewidth, and spectral weight.

As shown in Figs. R5 and R6, the inclusion of a phonon component — as well as variations in its spectral weight — does not alter the key characteristics of the magnetic excitations. Specifically: (1) Both the characteristic energy ω_0 and the peak energy ω_{max} of the paramagnon remain essentially unchanged within fitting uncertainties. (2) The linewidth of the magnetic peak remains nearly identical over the entire momentum range. (3) The momentum dependence of the magnetic spectral weight remains the same trend. Moreover, as shown in Suppl. Fig. 6, both the linewidth and spectral weight are comparable to those reported in other hole-doped infinite-layer nickelates [H. Lu et al., Science 373, 213 (2021), F. Rosa et al., PRB 110,224431 (2024), S. Fan et al., PRL 133, 206501 (2024)].

We therefore conclude that the possible contribution of phonon excitations has marginal influence on the key features of the paramagnon, and thus does not affect the conclusions of this work.

Corresponding revisions: We have included the above discussion as a new Suppl. Note 2B which includes Suppl. Figs. 2-4.

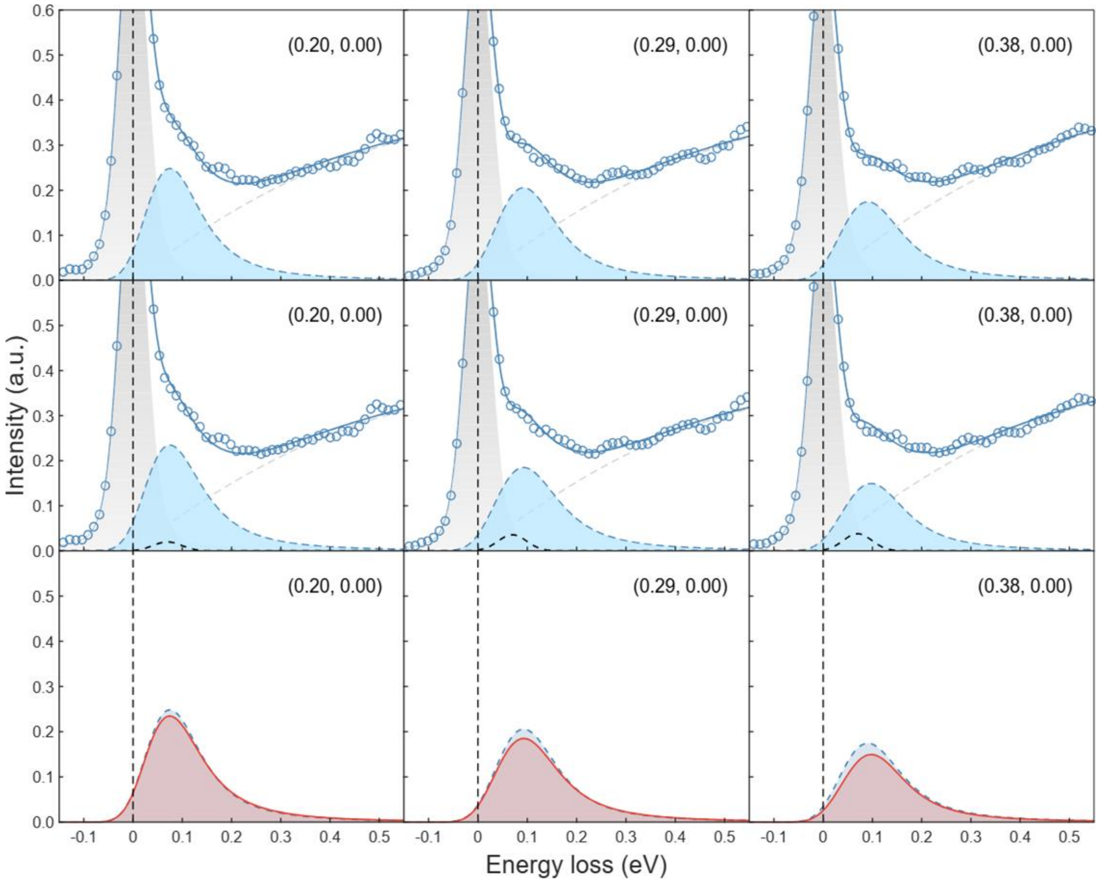

Fig. R5. Comparison of analysis with and without phonon contributions for OP SECNO. (Top row) Fittings of spectra using a model without and (middle row) with a phonon component at ~70 meV, indicated by black dashed curves. (Bottom row) magnetic spectral components extracted from the two fitting models. Red (blue) areas correspond to the model with (without) a phonon component. The dashed vertical lines indicate zero energy loss.

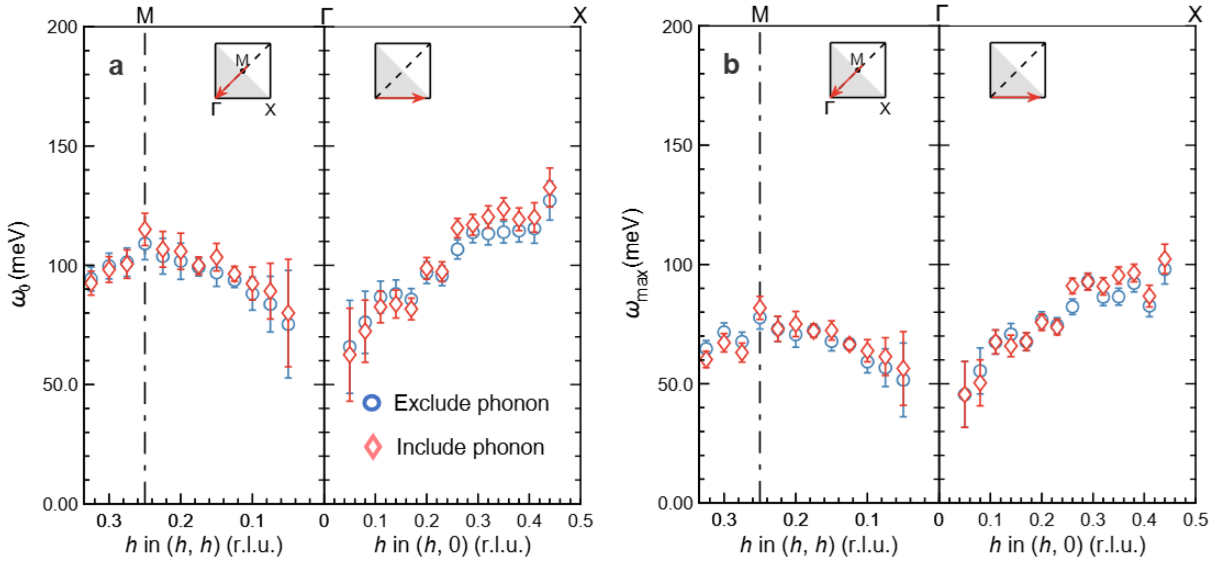

Fig. R6. Robustness of paramagnon dispersions against phonon contributions for OP SECNO. Dispersion of (a) the pole energy (ω_0) and (b) the peak maximum (ω_{max}). Results obtained using a fitting model without (blue circles) and with (red diamonds) a phonon component are compared.

I noticed that not all data points presented in Fig. 2 are included in Fig. 3e. It seems that all values below q=0.1 rlu are missing. There is probably a good reason for not considering this part of data, however the authors should justify their decision in the manuscript in order to be transparent.

Authors: We thank the referee for the careful notice. Indeed, the data points below q=0.1 r.l.u. shown in Fig. 2 are not included in Fig. 3e where we performed the spin-wave fitting. In Fig. R7(a), fitted paramagnon energies for all momentum points in Fig. 2 are plotted and included in a comparative spin-wave fitting. The excluded points near (0,0) show significantly large fitting errors. This occurs because, at such small in-plane momentum transfers, the scattering geometry approaches the specular condition, which drastically increases the elastic scattering — typical for soft X-ray RIXS. In addition, the paramagnon excitation shifts to lower energy, increasely overlapping with the elastic line. These two reasons in combination result in a larger uncertainty for the fitted paramagnon energy near (0,0).

For this reason, we restricted the quantitative analysis of the dispersion shown in Fig. 3e to momentum transfer q ≥ 0.1 r.l.u., where the magnetic excitations are better resolved. We stress that, as shown in Fig. R7, whether or not these points are included has very small influence on the fitted value of exchange coupling constants and does not affect the conclusions drawn from the comparison between different samples. In the revised manuscript, we have now explicitly clarified this point in the caption of Fig. 3, where we present the spin-wave fitting.

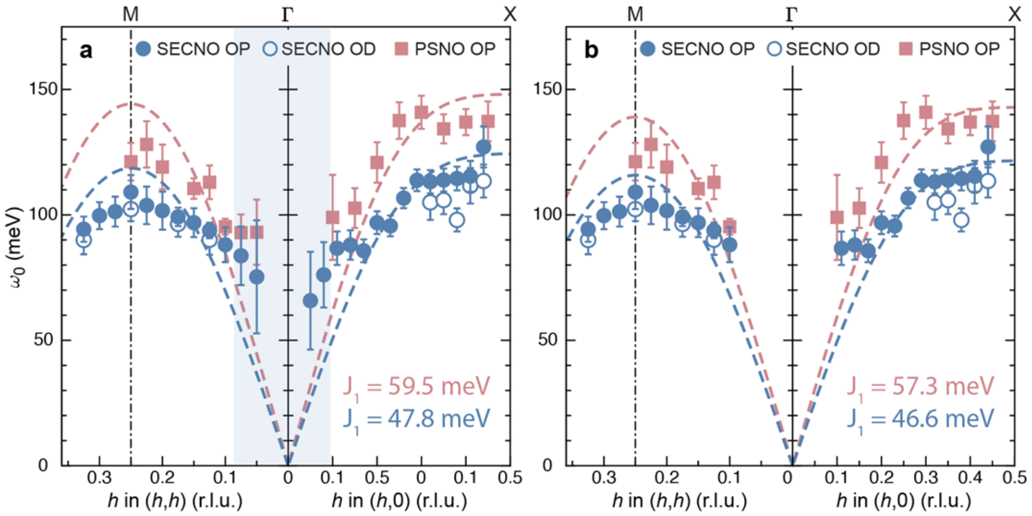

Fig. R7. Fitting results of paramagnon dispersions for SECNO and PSNO (a) with all momentum points measured and (b) excluding points close to specular condition [h ≤ 0.1 r.l.u. indicated by the light blue shade in (a)]. The resulting first-neighbour exchange couplings are given for OP PSNO (red) and OP SECNO (blue).

Another major point is that the presentation of the fitting results from the damped harmonic oscillator model is not complete. I am in particularly missing information on the susceptibility X’ and results on the damping constant γ. I would expect to see discussion of size of γ and comparison to findings from other infinite-layer nickelate compounds (see references in the Manuscript), which would be a critical part for discussion of robustness of the results. This data could be included in the Supplemental Information.

Authors: We agree with the referee that a more detailed discussion on the fitting results would further justify the robustness of our finding. Following this constructive suggestion, we now include a section in the Supplementary Information to discuss in detail the susceptibility χ' as well as the damping of the magnetic excitations.

As shown in Fig. R8a, the damping parameter γ of the paramagnon excitations extracted for both PSNO and SECNO are ~90 meV. To compare with other infinite-layer nickelates, we include a green shaded region in Fig. R8a to indicate the values of γ reported for other infinite-layer nickelates around optimal doping [H. Lu et al., Science 373, 213 (2021), F. Rosa et al., PRB 110,224431 (2024), S. Fan et al., PRL 133, 206501 (2024)]. Despite variations in experimental uncertainties and fitting procedures, the overall magnitude and momentum dependence are comparable. This agreement justifies the robustness of our results.

In order to assess whether the damped harmonic oscillator (DHO) model correctly extracts the imaginary part of the dynamical spin susceptibility $\chi''(q, \omega)$ and captures the essential characteristics of the magnetic excitation spectrum, we further conduct a model-independent spectral moment analysis and compare the results with those obtained from the DHO fitting. The n-th spectral moment is defined as $A_n(q)=\int\omega^n A(q,\omega)\,d\omega$, where $A(q,\omega)$ is the excitation spectrum. The first moment A_1 naturally represents the average excitation energy, while the square root of the second moment ($A_2=\int(\omega-A_1)^2A(q,\omega)\,d\omega$) characterizes the energy spreading of the spectrum around this average

value. Additional details of the spectral moment analysis are given in Suppl. Note 2D. In both systems, results from the spectral moment analysis are fully consistent with the DHO fitting, with the first and second moment satisfying A_1(q) ≈ ω_0 (compare with Fig R4a) and √A2(q) ≈ γ, respectively. This demonstrates that our DHO modeling provides a reliable and physically meaningful description of the magnetic excitations for both systems.

Corresponding revisions: We have included a new Supplementary Note 2D and Suppl. Fig. 6 to discuss the results of the spectral moment analysis.

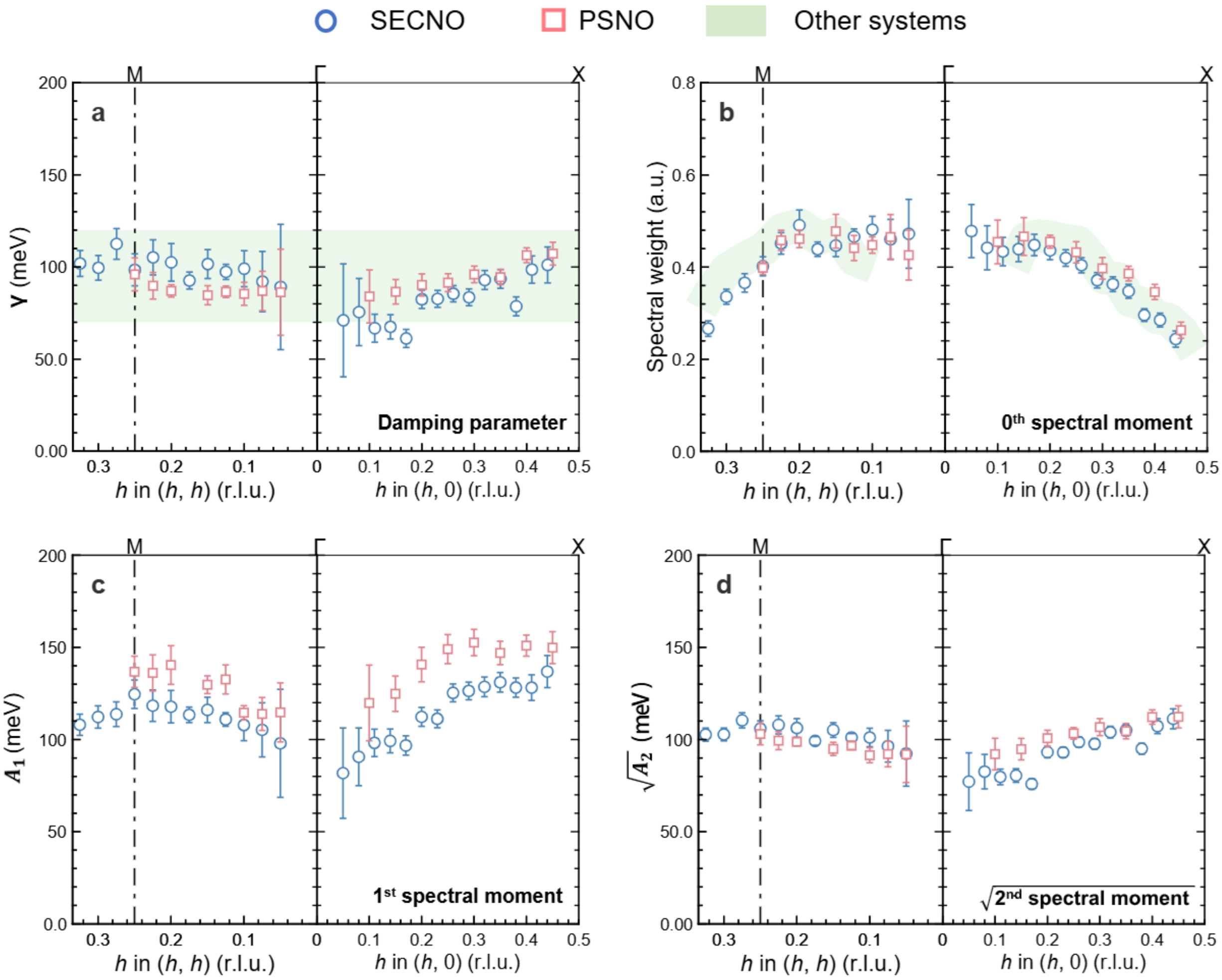

Fig. R8. (a) Damping parameter γ_q extracted from the DHO fitting for optimally doped SECNO and PSNO, the light green shaded region indicates the range of γ values reported for other infinite-layer nickelate systems around optimal doping [H. Lu et al., Science 373, 213 (2021), F. Rosa et al., PRB 110,224431 (2024), S. Fan et al., PRL 133, 206501 (2024)]. (b-d) $A_0(q)$, $A_1(q)$ and $\sqrt{A_2}(q)$, respectively. The zeroth moment $A_0(q)$ in (b) is normalized to the intensity of the *dd* excitations. The light green shaded line in (b) outlines the trend of spectral weight in $Nd_{0.775}Sr_{0.225}NiO_2$ adapted from H. Lu et al., Science 373, 213 (2021).

Indications for softening of magnetic excitations in infinite-layer nickelates was reported already in previous studies and therefore the authors should cite this work when discussing their own results in Lines 175-182 and 206-213 (see H. Lu et al. and F. Rosa et al., mentioned above). At the same time, contrary to the manuscript, this topic is still controversial for the cuprate systems. Besides reports of softening of magnetic excitations with doping, there are publications claiming opposite behavior (see Y. Y. Peng, Phys. Rev. B 98, 144507 (2018) and H. C. Robarts, Phys. Rev. B 100, 214510 (2019)).

Authors: We thank the referee for pointing out the relevant references. We have now cited them when mentioning the doping-induced softening of magnetic excitations in the revised manuscript. We also agree on the existence of controversy for magnon softening in cuprates, and have therefore softened our statement about cuprates. The revised sentences now reads: "*This trend is consistent with the doping evolution reported in other nickelate systems. Such behaviour indicates a gradual softening of spin correlations as additional carriers are introduced, resembling the observations reported in certain hole-doped cuprates.*"

Further remarks, where described effects could be better specified/ quantified:

- Lines 140-142: no detailed comparison between crystal field in Sm-based and Nd-based compounds is presented, therefore it cannot be claimed that the crystal field environment is similar. However, the authors might state that the dd excitations don't show obvious differences.

Authors: We thank the referee for the suggestion and have revised the statement accordingly.

- Lines 148-149: how does the resonant behavior demonstrate the electronic origin? (what would be an alternative origin?)

Authors: The behavior of a signal resonating with the Ni absorption edge demonstrates that it is related to the valence states of Ni orbitals. Inelastic signals from alternatively origins, for example, purely structural effects are not expected to exhibit such a resonant behavior. Based on the referee comment, we have improved this sentence such that it now reads: "*The resonant behavior demonstrates its electronic origin, arising from valence-state transitions of Ni 3d orbitals.*"

- Lines 163-166: increasing energy with increasing momentum is not sufficient to claim characteristic dispersion of square-lattice antiferromagnetic paramagnons, but rather shows an agreement.

Authors: We agree with the referee and have revised the statement "*displaying the characteristic dispersion of a square-lattice antiferromagnetic paramagnon*" into "*in agreement with the characteristic dispersion of a square-lattice antiferromagnetic paramagnon*".

- Lines 171-172: how broad is the width of paramagnons?

Authors: The energy width of the paramagnons is about 170 meV (~2γ). This information is now included in this sentence, which is now changed to "*The broad energy width (~170 meV) — significantly exceeding the resolution — further supports our assignment of this excitation as a paramagnon.*"

- Lines 172-174: from the presented data I do not see that the spectral weight is suppressed and the linewidth broadens (with increasing momentum). In case this finding is important, the authors should provide a graph showing these effects and quantify them.

Authors: The quantitative analysis results of linewidth parameter γ and spectral weight are now

provided in the new Suppl. Fig. 5, along with the results from spectral moment analysis.

Remarks related to figures, partially due to missing information:

- At which momentum transfer was data in Fig. 1 it measured? Alternatively, a table/graph relating the incidence angle with momentum transfer could be provided in the Supplementary Information.

Authors: RIXS data in Fig. 1 were measured at Q = (0.35,0,0.31). XAS data were measured at an incident angle of 90° (normal-incidence). We have now revised the figure caption to include this information. Following the referee's suggestion, we also included a Suppl. Table 2 to list momentum transfer values (h,k,l) and corresponding rotation (scattering, incident, sample azimuthal) angles for measured spectra.

- At which photon energy were spectra presented in Fig. 2 and 3 obtained?

Authors: RIXS data presented in Figs. 2 and 3 are obtained at the Ni L_3 resonance with a photon energy of 847.5 eV, which maximizes the intensity of the low-energy excitations. This information is now included in both figure captions.

- At which momentum transfer was each line plot in Fig. 2c measured?

Authors: We have revised Fig. 2c to indicate in-plane momentum transfers for selected line plots. A complete list of scattering geometry and corresponding momentum transfer (h,k,l) for each spectrum is provided in the new Suppl. Table 2. The information of momentum transfer is also indicated in the new Suppl. Fig. 7.

- The color-scale in Fig. 2 d,3 is not favorable, since there is little contrast between yellow, white and blue.

Authors: We have replaced the original color scale with a high-contrast scheme ranging from black through green and yellow to red.

- More horizontal tics for all graphs in Fig. 3 would be useful to follow the data.

Authors: More horizontal tics are now added to Fig. 3. We thank the referee for this suggestion.

- Indicating size of energy resolution as horizontal line either in the false-color plot or in the dispersion graphs would be informative, as it presents a limitation to the sensitivity of the data

Authors: In the revised Fig. 3e (dispersion plot), we now indicate the energy resolutions (FWHM), which are well below the paramagnon bandwidth (ω_0 at zone boundary). With significant counting statistics, such resolutions are sufficient to resolve the difference in paramagnon dispersions between the two nickelate systems.

Reviewer #3 (Remarks to the Author):

Yan and coworkers are reporting a RIXS study on the magnetic excitations of some nickelate superconductors. In particular, the authors find that paramagnons persist in optimally doped (OP) and even overdoped (OD) Sm-based infinite-layer (IL) nickelate SCs with a Tc up to 40K. Most importantly, they report that the peak energy of the magnons of the OP sample with Tc of 40K is lower than for corresponding Pr-based IL-nickelates SCs with a lower Tc of only 10K. This suggests

a negative correlation between Tc and the in-plane exchange interaction J that is different from the cuprates and therefore of interest for researchers from within the high Tc community. Their suggestions that multi-band effects due to hybridization between rare earth 5d and Ni 3d orbitals levels and/or Kondo-correlations due to enhanced c-axis coupling may need to be considered, will certainly motivate further experimental and theoretical work along these lines. However, I am not quite sure whether their results and conclusions will be of sufficient interest for researchers beyond the high Tc community. I am therefore reluctant to propose this manuscript for a journal like Nature Communications.

Authors: We thank the reviewer for the thoughtful and constructive evaluation of our manuscript. We appreciate the recognition of the physical significance of our findings and fully agree that the persistence of paramagnons in infinite-layer nickelates is primarily of interest to the high-T_c community. Nevertheless, we would like to emphasize that our results also bear broader implications for understanding correlated-electron physics and unconventional superconductivity beyond the specific nickelate family.

Our results specifically show that, unlike in cuprates, there is no positive correlation between the superconducting transition temperature T_c and the in-plane exchange coupling $J_{//}$ in infinite-layer nickelates. This contrast offers a new view on how magnetic exchange interacts with Cooper-pair formation, implying that the pairing mechanism might change as the system shifts from single-band cuprates to multi-band nickelates.

Moreover, the possible involvement of hybridization between rare-earth 5d and Ni 3d states, and Kondo-like coupling points toward an intertwining of itinerant and localized degrees of freedom. Such physics is relevant not only to nickelates but also to a much broader class of multi-orbital correlated materials, including heavy-fermion compounds [G. R. Stewart, Rev. Mod. Phys. 56, 755 (1984)] and other systems [e.g., B. Xu et al., PNAS 121, e2401430121 (2024), R. Lou et al., PRL 135, 146902 (2025)] where orbital hybridization strongly renormalizes the electronic structures and magnetic interactions. Hence, our work connects the phenomenology of infinite-layer nickelates to broader questions concerning how hybridization and dimensionality effects shape magnetic excitations and superconductivity.

We note that *Nature Communications* has previously featured studies that focus on a particular material class yet introduce conceptual advances of broader relevance — for instance, in cuprates [Q. Wang et al., *Nat. Commun.* 13, 1795 (2022)], ruthenates [Suzuki H. et al., *Nat. Commun.* 14, 7042 (2023)], and in nickelates [Shi, M., et al., *Nat. Commun.* 16, 9141 (2025)]. In a similar spirit, we believe our results provide new insights into the nature of spin correlations and their relationship to high-temperature superconductivity, appealing to researchers across the broader condensed-matter community.

Moreover, I have some major questions regarding the presentation and interpretation of the data.

Authors: We appreciate the reviewer's thoughtful comments and provide responses to each point below.

Most importantly, there appears to be a large offset in the energy of the peak intensity in the maps of Fig. 2d-e and the magnon peak energies shown in Fig. 2c and Fig. 3. I wonder what causes this large difference in peak energies by almost a factor of two? Is it due to a mistake in labeling the y-scale in Figs. 2d and 2e? Otherwise, how can it be that the intensities peak at such difference energies in Fig. 2c and Fig. 2d?

Authors: We thank the reviewer for pointing this out. Indeed, there was a misplacement of the label in the original version of Figs. 2d,e — the zero point on the energy axis was misplaced during the vector-graphic export process. We have corrected this in the revised Fig. 2.

The authors mention that a possible explanation for their finding, that Tc does not scale with the in-plane J, is the hybridization with the rare earth ions and a subsequent enhanced 3D character of the Sm based system. This raises the question how this would affect their analysis of the RIXS data where it seems to me that the out-of-plane component of the scattering vector has not been considered?

Authors: We thank the referee for this comment. We have added Suppl. Fig. 8 (Fig. R2), where the out-of-plane component of the scattering vector is explicitly provided for each measured point. Due to the constraints from scattering geometry, the accessible range of L is limited to ~0.2 to 0.4 r.l.u. (see a complete list in Suppl. Table 2). In addition, we have performed a comparative spin-wave modeling including a finite interlayer exchange interaction $J_{\perp}$, to assess the possible influence of out-of-plane modulation on our conclusions.

With a finite $J_{\perp}$ a gap will occur at (0,0,L) with the size depending on the value of L and $J_{\perp}$. However, it is challenging to resolve such a gap due to the damping of the paramagnon and the increasing elastic scattering when approaching specular condition at (0,0,L). Nevertheless, as shown in Fig. R2 and Suppl. Fig. 8, the comparative analysis demonstrates that while $J_{\perp}$ sets the size of the gap, it has marginal influence on the extracted in-plane $J_{//}$ which is primarily determined by the zone-boundary energy. We would like to emphasize that although the reduced in-plane $J_{//}$ in Sm-based system hints at an enhanced three dimensionality in magnetic interactions compared to other IL nickelates, the in-plane exchange couplings are still expected to dominate, as suggested by theoretical calculations in Y. Zhang et al., PNAS Nexus, 2, pgad108 (2023).

As such, the three-dimensionality effect does not influence the evaluation of the in-plane exchange couplings and our conclusion on its absence of scaling with T_c.

Corresponding revisions: We have added the above analysis and discussions in Suppl. Note 3, and included Fig. R2 as Suppl. Fig. 8.

Another technical question. The SECS and PECS samples have been measured at different instrumental setups with different energy resolutions. This makes me wonder how the authors can be sure that the lower energy resolution of the SECS data does not lead to an apparent reduction of the fitted peak energy?

Authors: We thank the reviewer for raising this important technical question. Indeed, the SECNO and PSNO samples were measured under slightly different experimental conditions, with energy resolutions of 67 meV and 46 meV, respectively. However, this difference does not affect the reliability of the fitted peak positions.

This is because, in the doped nickelates, the magnetic excitations are significantly damped. The intrinsic linewidth is described by the damping parameter γ, which is much larger than the experimental energy resolution. For example, for optimally doped SECNO and PSNO, $2\gamma \approx 170$ meV, while the instrumental resolution is below 70 meV. Therefore, the peak profiles are determined by the intrinsic response, rather than limited by resolution. To justify this, we have conducted a spectral moment analysis as shown in Suppl. Fig. 6. The results clearly show that magnetic excitations in optimally doped SECNO and PSNO exhibit comparable damping and spectral weight, while the excitation energy is lower in SECNO, as determined from both the DHO fitting and the first spectral

moment analysis.

Corresponding revisions: To demonstrate this, we have now provided more details on both energy and momentum resolutions in Methods. Additional spectral moment analysis is included as Suppl. Note 2D and Suppl. Fig. 6.

A related question. In the technical part on the RIXS experiments the authors state that "The beamline-specific photon energy offset has not been considered". I do not understand what exactly this means. Have they shifted the energy scales to match the positions of the elastic peaks? Otherwise, how can they be sure that the shift of the magnon peak energy is not caused by different energy offsets of the spectrometers?

Authors: We thank the reviewer for the opportunity to clarify this point. The term *"beamline-specific photon energy offset"* refers to the _minor systematic variation in the absolute photon-energy calibration across beamlines_, arising from variations in monochromator optics or reference calibration procedures, which typically exists for a RIXS beamline.

In our RIXS analysis, however, we focus on the energy-loss value ($E_{loss}=E_{in}-E_{out}$), where the elastic peak of each spectrum is aligned to zero energy loss. This standard procedure effectively removes any beamline-dependent photon-energy offset, ensuring a consistent zero-energy reference across all datasets. Therefore, the statement "*not considered*" means that no additional artificial correction was introduced beyond this elastic-peak alignment, since the energy-loss calibration is already internally consistent. The differences in magnon peak energies between SECNO and PSNO are thus intrinsic and not caused by spectrometer or photon-energy offsets.

Corresponding revisions: We have now clarified this point in the revised Methods section. The revised sentence now reads: "_The zero energy loss is calibrated using the position of the elastic peak, ensuring that it remains unaffected by minor systematic variations in the absolute photon energy calibration across different beamlines._"

Minor comments.

In the cuprates the rare earth can also matter, i.e. see Pr doped YBCO for which Tc and metallicity is suppressed.

Authors: We agree with the referee's comment. When f-electrons of Pr are involved in the low-energy electronic structure (as is the case of $PrBa_2Cu_3O_7$ and $PrFe_4P_{12}$), it will significantly influence the electronic properties. To be more rigorous, we have softened the claim and rephrased the corresponding expression into "_Whereas rare-earth elements do not exhibit a pronounced influence on the electronic structure of most cuprates,..._".

I suggest that the references concerning the positive correlation between Tc and J in the cuprates should be given already in the introduction, i.e. not only at the final discussion.

Authors: We thank the referee for the suggestion and have now cited the relevant references at the end of the introduction, when first mentioning the trend in cuprates.

Finally, I find the abbreviations SECS and PSNO somewhat inconsistent, i.e., why is the oxygen (O) included for the latter but not the former system?

Authors: Previously, we followed the abbreviation SECS used in the work [Nature 642, 58 (2025)] reporting the discovery of superconductivity in this system, which did not include nickel and oxygen to avoid being lengthy. In fact, the optimally- and over-doped samples used in our study are co-doped only with Eu and Ca but without Sr. For consistency, we have now changed the abbreviation of the Sm-system into SECNO throughout the manuscript.

We would like to thank all the referees once again for their insightful comments, which have significantly improved our manuscript.

(END)

Reply to Reviewer Comments

Reviewer #1 (Remarks to the Author):

The authors reasonably addressed my questions, as well as those of the other reviewers, and revised both the manuscript and the supplementary material.

Authors: We thank the reviewer again for the constructive comments that have helped to improve our manuscript.

Reviewer #2 (Remarks to the Author):

I would like to thank the authors for addressing my comments. The authors now show more details on the measured spectra in the supplement. Nevertheless, I believe that the new version of the manuscript still needs improvements:

Authors: We appreciate the reviewer's additional careful evaluation of our revised manuscript. Below we provide a point-by-point response to the reviewer's remaining concerns and describe the corresponding changes made in the revised version.

1) I would recommend that the authors display an overview with all analyzed RIXS line spectra in the Supplementary information (as done e.g. in Ref. [7]).

Authors: We thank the reviewer for this suggestion. In the revised Supplementary Information, all analyzed RIXS line spectra are now displayed in the new Supplementary Fig. 9. A complete list of measurement geometry for each spectrum is given in Supplementary Table 2.

2) Comparing Fig. R7 a) and b) I don't see a reason why the data points close to the Gamma point should not be shown in the Manuscript. They have larger error bars than the data points for larger momentum transfer, however the fitting results are in both cases similar and within the error bars. Therefore, I would recommend to replace Fig. 3 e) with Fig. R7 a) (without the blue shaded area), which includes all data points.

Authors: This is a helpful suggestion. In the revised manuscript, we have included data points close to the Gamma point in Fig. 3(e), Supplementary Figs. 7 and 8. The corresponding fitting results are updated throughout the manuscript (now using error-bar-weighted fitting), while the conclusions remain unchanged. For clarity, we also provide an explanation in the figure caption for the increasing error bars upon approaching the Gamma point: "Larger error bars near (0,0) stem from increasing elastic scattering around the specular geometry (See Supplementary Fig. 9)."

3) Lines 169-182: this section is not written in a very coherent way. On one side the authors still discuss the origin of the observed 0.1 eV excitation and at the same time compare already details of their results with other systems. The two should be separated. Also, the discussion on possible

non-isotropy of magnetic excitations in SECNO can be ideally moved to later section, where modelling of the effective exchange interactions is discussed.

Authors: We thank the reviewer for this valuable suggestion regarding the organization and clarity of this paragraph. In the revised manuscript, this paragraph now first focuses on describing the characteristic of the ~0.1 eV excitation and on establishing its origin. We have moved the comparison with other IL nickelates to the end of the paragraph.

The revised sentence now reads: "The overall dispersive behaviour, the momentum dependence of the spectral weight, and the energy linewidth of the magnetic excitations in OP SECNO are consistent with previous observations of spin excitations in other IL nickelates"

The discussion on the possible non-isotropy of the magnetic excitations is now moved to the later section where we model and discuss the effective exchange interactions.

4) Lines 183-193: I find the statement of a general trend too strong. The authors should rather say that two samples with different hole doping were studied and that the energy of magnetic excitations of the sample with higher doping is very likely lower (this is what can be seen from Fig. 3.). Later, in the discussion part, comparison to other nickelate systems and cuprates shall be made, where it can be argued that the presented observations from SECNO points toward similar trends.

Authors: Following the reviewer's suggestion, we have softened our statement on the doping evolution of the magnetic excitations in SECNO. The revised statement now reads:

"The paramagnon persists in the overdoped SECNO sample. Compared with the optimally doped sample, the overdoped sample shows a likely reduction in both the pole energy ω_0 (undamped excitation energy, see Methods and refs. 52 and 53) and the energy positions of the peak maxima (ω_{max}) (see Fig. 3e and Supplementary Fig. 1). While our measurements are limited to these two dopings, this observation is consistent with the doping evolution reported in other nickelate systems."

In fact, I am a puzzled when comparing Fig. 3e with Fig. S1, since the differences in the two displayed RIXS spectra in the later seem to be much larger than the results presented in Fig. 3e. What explains this deviation? Besides the two graphs, there is no other data presented for the overdoped sample, neither complete analysis results.

Authors: To facilitate comparison with results from other IL nickelates (e.g., Ref. 7), we plotted in Fig. 3e the paramagnon pole energies ω_0, i.e., the undamped excitation energy. As mentioned in the Methods section and shown in Ref. 52, the difference between ω_0 and the energy positions of the peak maximum (ω_{max}) increases with damping. In the revised Supplementary Fig. 6, it is shown that the damping increases slightly in the overdoped sample; consequently ω_{max} shifts further to lower energies, which is directly visible in Fig. S1.

We agree that this point was not clearly communicated. To improve this, we now include all analyzed RIXS spectra for the overdoped SECNO sample in the new Supplementary Fig. 9. The corresponding

fitted damping parameters are summarized in Supplementary Fig. 6, and the extracted pole energies are presented in Fig. 3. We thank the reviewer for this helpful comment.

5) Lines 217-219: In my opinion this sentence can be moved to the results section. What are "extended defects" and why are the samples free of such defects?

Authors: Different from point defects, "extended defects" refer to defects that extend over several lattice spacings. In infinite-layer nickelates, such defects are usually formed by Ruddlesden-Popper-type faults due to the imperfect topotactic reduction process, which can be identified using STEM. This has been discussed in previous studies [e.g., Li et al., PRL 125, 027001 (2020)]. Similar to the report in Lee et al., Nature 619, 288 (2023), by carefully choosing the substrate and controlling the reduction conditions, our SECNO samples are shown to be free from such defects, as demonstrated by the characterization in Chow et al., Nature 642, 58 (2025). For clarity, the revised manuscript now describes these defects explicitly as "Ruddlesden-Popper-type extended defects".

Following the reviewer's suggestion, we have moved the sentence (previously in Lines 217-219) to the beginning of the results section, which improves the logical flow.

6) Lines 219-224: Can be merged with discussion of possible softening of magnetic excitations with doping for SECNO. However, in my opinion this paragraph does not fit well in between the previous and the following paragraph and should be moved somewhere else.

Authors: We have now merged this part with the discussion of possible doping induced softening of magnetic excitations for SECNO.

7) Lines 275-277: which particular differences are meant here?

Authors: The particular differences here refer to those directly relevant to our discussion, including: (i) the potentially more significant contribution of rare-earth electronic states at low energy and (ii) the stronger interlayer coupling in the IL nickelates suggested by our results.

In the revised manuscript, we now specifically highlight these potential differences here. The revised sentence reads: "The potentially enhanced contribution of rare-earth electronic states at low energy and the stronger interlayer coupling in IL nickelates, compared to cuprates, could be fundamental for understanding their superconducting mechanisms."

8) Lines 277-281: what are the alternative design principles for superconducting nickelates and cuprates?

Authors: Motivated by our findings, a possible route to enhanced superconductivity in infinite-layer nickelates is to further strengthen the interlayer coupling. This could be pursued, for example, by applying epitaxial strain and/or hydrostatic pressure, or via heterostructure engineering. In the revised manuscript, we now include these directions as an outlook.

By implementing the reviewer's suggestions, we believe that the revised manuscript now meets the publication standards of *Nature Communications*. We appreciate the reviewer's comments that have helped further improve the clarity of this work.

Reviewer #3 (Remarks to the Author):

The authors have responded well to the questions and suggestions that I had raised in my previous report. In particular, they have answered well to all my technical questions/reservations. Moreover, they have provided rather a convincing discussion of why their results could be of interest for a broader research community, and not just the experts in the particular field of high-Tc superconductors.

I am therefore inclined to agree with the authors that this manuscript is suitable for publication in Nature Communications.

Authors: We thank the reviewer for the positive assessment of the revised manuscript, and appreciate the reviewer's time in reviewing the manuscript again.

(END)

Reply to Reviewer Comments

Reviewer #2 (Remarks to the Author):

The authors have addressed all my questions. I have no further comments.

Authors: We thank the reviewer for the positive assessment of the revised manuscript, and appreciate the reviewer's time in reviewing the manuscript again.

(END)
